# Recovery of a learned behavior despite partial restoration of neuronal dynamics after chronic inactivation of inhibitory neurons

**DOI:** 10.1101/2023.05.17.541057

**Published:** 2023-12-15

**Authors:** Zsofia Torok, Laura Luebbert, Jordan Feldman, Alison Duffy, Alexander A. Nevue, Shelyn Wongso, Claudio V. Mello, Adrienne Fairhall, Lior Pachter, Walter G. Gonzalez, Carlos Lois

**Affiliations:** 1 Division of Biology and Biological Engineering, California Institute of Technology; Pasadena, CA, USA.; 2 Department of Computing and Mathematical Sciences, California Institute of Technology; Pasadena, CA, USA.; 3 University of Washington; Seattle, WA, USA.; 4 Oregon Health & Science University; Portland, OR, USA.; 5 Department of Physiology, University of San Francisco; San Francisco, CA, USA.

## Abstract

Maintaining motor skills is crucial for an animal’s survival, enabling it to endure diverse perturbations throughout its lifespan, such as trauma, disease, and aging. What mechanisms orchestrate brain circuit reorganization and recovery to preserve the stability of behavior despite the continued presence of a disturbance? To investigate this question, we chronically silenced a fraction of inhibitory neurons in a brain circuit necessary for singing in zebra finches. Song in zebra finches is a complex, learned motor behavior and central to reproduction. This manipulation altered brain activity and severely perturbed song for around two months, after which time it was precisely restored. Electrophysiology recordings revealed abnormal offline dynamics, resulting from chronic inhibition loss, some aspects of which returned to normal as the song recovered. However, even after the song had fully recovered, the levels of neuronal firing in the premotor and motor areas did not return to a control-like state. Single-cell RNA sequencing revealed that chronic silencing of interneurons led to elevated levels of microglia and MHC I, which were also observed in normal juveniles during song learning. These experiments demonstrate that the adult brain can overcome extended periods of abnormal activity, and precisely restore a complex behavior, without recovering normal neuronal dynamics. These findings suggest that the successful functional recovery of a brain circuit after a perturbation can involve more than mere restoration to its initial configuration. Instead, the circuit seems to adapt and reorganize into a new state capable of producing the original behavior despite the persistence of some abnormal neuronal dynamics.

## Introduction

Maintaining the ability to precisely execute behaviors throughout life, despite perturbations due to trauma, disease, or aging, is crucial for reproduction and survival. To reliably execute behaviors, brain circuits require a balance of excitation and inhibition (E/I balance) to maintain physiological activity patterns. Loss of E/I balance causes abnormal patterns of neuronal activity, which can result in diseases such as epilepsy^1,2^. Given its importance, brain circuits strive to restore E/I balance once disturbed^3^. However, the characteristics of circuit reorganization and recovery of E/I balance after extensive and long-term perturbation remain poorly understood.

Zebra finches produce a highly stereotyped song with minimal variability over extended periods of time^4^, underpinned by temporally precise neural activity^5^. This species thus provides an ideal model to simultaneously track changes to abnormal brain activity and its effect on behavior over time. Here, we genetically blocked inhibitory neurons in HVC (proper name), a premotor brain nucleus of the male zebra finch involved in song production, to chronically perturb the E/I balance. HVC contains two main types of excitatory neurons that project to two main downstream targets: nucleus X (proper name) and nucleus RA (robust nucleus of the arcopallium). In addition, HVC includes several types of inhibitory neurons whose axons do not leave HVC; thus, they act locally within HVC^5^. Previous work has shown that acutely blocking interneuron signaling in adult finches by chemicals perturbs behavior for a few hours until the chemical is washed out, after which normal behavior is restored^6^. These transient perturbations, however, offer limited insight into the recovery response of neural circuits, as the restoration of brain function and behavior occurs within a few hours after the perturbation ceases. Although previous studies have demonstrated the transient effects of acutely blocking interneuron signaling in adult animals^6^, the long-term impact on neuronal dynamics, circuit structure, and the potential for behavior and brain state recovery remain unexplored. To investigate this question, we chronically silenced a fraction of inhibitory neurons in the HVC nucleus of adult zebra finches, which altered brain activity and severely perturbed their songs for around two months. Electrophysiology recordings revealed that song degradation was paralleled by abnormal offline activity; however, subsequent recovery of the behavior occurred despite incomplete normalization of brain activity. Persistent suppression of interneuron activity resulted in upregulation of microglia and major histocompatibility complex class I (MHC I) expression during the peak of behavioral distortion, suggesting potential involvement of inflammatory and immune signaling pathways in neural circuit restructuring. Interestingly, microglia levels reverted to baseline upon the onset of behavioral recovery, indicating a dynamic relationship between neuroinflammatory responses and circuit restoration. These experiments demonstrate that the adult brain can recover after extended periods of drastic abnormal activity and reveal some of the cellular mechanisms that potentially mediate functional restoration after brain perturbation.

## Results

To investigate how a complex behavior recovers after chronic loss of inhibitory tone, we permanently blocked the function of HVC inhibitory neurons in adult male zebra finches by bilateral stereotaxic injection of an AAV viral vector into HVC. The AAV viral vector carried the light chain of tetanus toxin (TeNT), driven by the human dlx5 promoter, which is selectively active in inhibitory neurons^7^. TeNT blocks the release of neurotransmitters from presynaptic terminals, thereby preventing neurons from communicating with their postsynaptic partners^8^. Thus, the expression of TeNT does not directly alter the ability of neurons to fire action potentials but effectively mutes them. As a control, a second group of animals was injected with an AAV carrying the green fluorescent protein NeonGreen driven by the ubiquitous promoter CAG. Throughout various time points in this perturbation paradigm, we recorded songs, obtained electrophysiological measurements (chronic and acute within HVC), and measured changes in gene expression at single-cell resolution.

### Permanent genetic muting of inhibitory neurons in HVC leads to zebra finch song degradation and subsequent recovery

**Figure 1:**
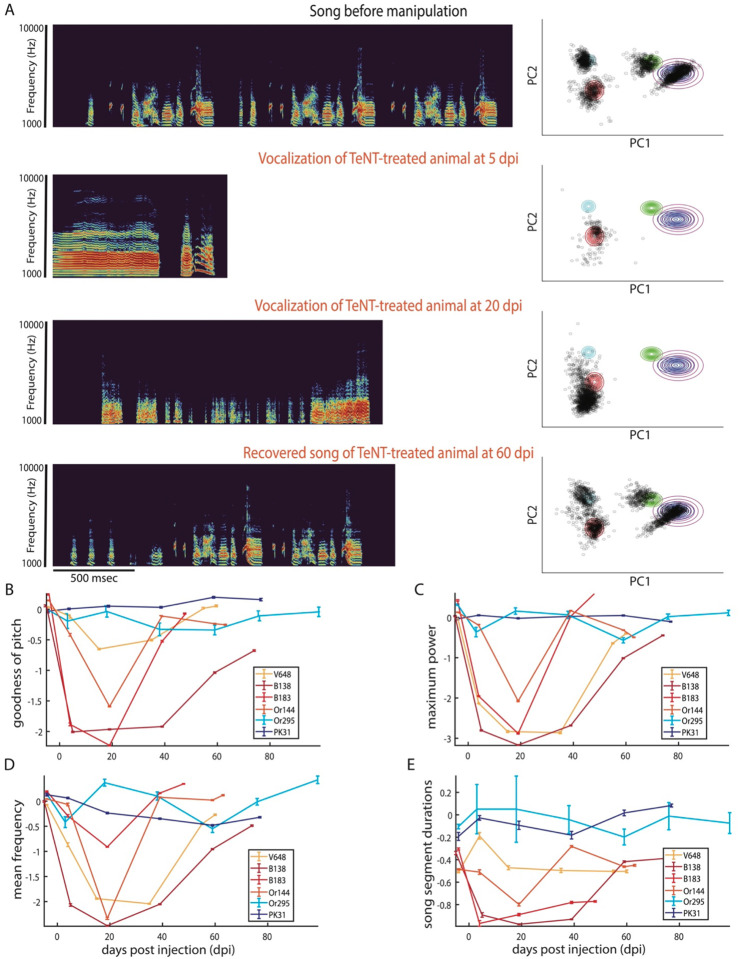
Song degradation and recovery after chronic inactivation of inhibitory neurons in HVC by viral expression of tetanus toxin (TeNT). **A** (**left)** Examples of spectrograms of songs produced by an animal treated with TeNT before, and 5, 20 and 60 days post- injection (dpi). Data from this animal (V648) are also shown in B-E. **A (right)** 2D principal component analysis (PCA) visualization of syllables aka song segments produced by the animal on the same day as the spectrogram example on the left. Note: even when the songs are degraded, we continue to refer to an uninterrupted length of vocalization as a ‘syllable or song segment’. Individual dots indicate single syllable renditions. In the unperturbed song (before virus injection), syllables cluster into distinct groups. See Methods for description of acoustic features used in the PCA space. **B-E** Different features of the vocalizations before and after stereotaxic injection of control (GFP-expressing) or TeNT virus. The mean frequency and goodness of pitch measures represent averages over song segments. Control animals (Or295 and PK31) are labeled in shades of blue (n=2); TeNT-treated animals (V648, B183, B138 and Or144) are labeled in shades of red and orange (n=4). Traces indicate averages over syllables sung within the 3 closest days of recorded vocalizations. Error bars indicate standard error. Values are normalized relative to distributions 5 days before perturbation (see Methods).

An average male zebra finch song contains 4 to 7 units called syllables. These syllables are fixed in order and make up a motif repeated multiple times to form a bout^9–11^. A bout defines the length of a vocally active period in adult animals. Throughout all experiments, the songs of the individually housed animals were continuously monitored from 7–20 days pre-injection to 60–100 days post-injection (dpi) using sound-isolated chambers. In control animals, the song did not change at any time after the viral injection. For the first 24–48 hours after injection, the song of TeNT-treated animals was virtually identical to their original song. However, starting at 2–5 dpi, the song of TeNT-treated animals began to degrade, both in its acoustic and temporal features (Figure 1). By 10 dpi, the song of TeNT-treated animals was highly degraded and bore no resemblance to the original song before viral injection (Figure 1 A).

During the first few days of song degradation, TeNT-treated animals produced instances of syllables with abnormally long durations far outside the range of any adult syllable duration observed in control animals (Figure 1 A shows one such vocalization at 5 dpi; examples of syllable length distribution are shown in Supplementary Figure 1). Later (8 to 30 dpi), all vocalizations became very short (< 100 msec) with inter-syllable intervals of varying lengths (100 msec to 1 sec) between them (spectrogram examples in Supplementary Figure 2). At 20–30 dpi, the song of TeNT-treated animals was highly variable between renditions and lacked the normal organization of motif and bout (Figure 1 A). However, by 40–50 dpi, the structure of the song started to re-emerge. By around 70 dpi, the song of TeNT-treated animals was highly similar to the song produced before viral injection (Figure 1).

### Chronic electrophysiological recordings reveal that the degree of song distortion is correlated with the presence of large-amplitude voltage deflections in HVC during nighttime

To investigate the electrophysiological changes in HVC that accompany song degradation due to permanent loss of inhibition and subsequent behavioral recovery, we continuously recorded electrical activity in HVC across the entire duration of song degradation and recovery (0–90 dpi).

**Figure 2:**
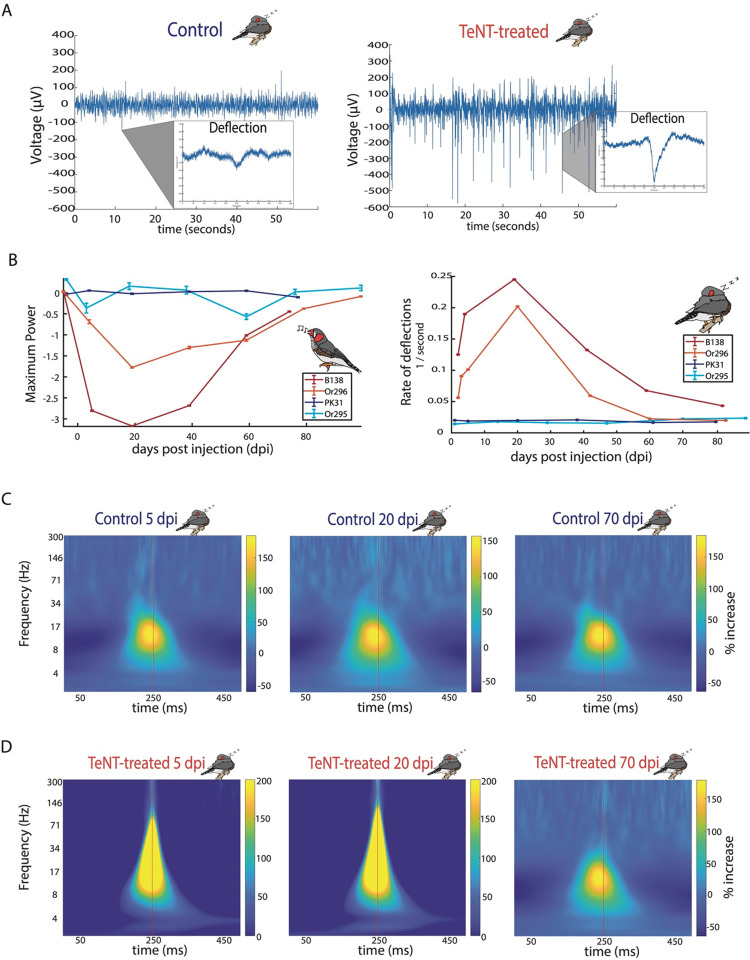
Chronic electrophysiology recordings within HVC after chronic inactivation of inhibitory neurons by viral expression of TeNT. **A** Raw electrophysiology traces during night time (lights off period) from TeNT-treated and control animals 5 days post-injection (dpi). Note: electrodes were implanted on the same day of viral injection. Insets show examples of stereotypical deflections that at 5 dpi were approx. 50 ms in duration, and −530 μV in amplitude, in TeNT-treated animals, compared to 96 ms and −140 μV in control animals. **B (left)** Quantification of song quality of chronically implanted animals over time. Control animals (PK31 and Or195) are depicted in shades of blue. TeNT-treated animals (B138 and Or296) are depicted in shades of orange and red. **B (right)** Rate of deflection events during night time. Control animals (PK31 and Or195) are depicted in shades of blue. TeNT-treated animals (B138 and Or296) are depicted in shades of orange and red. The highest rate of sleep voltage deflections in TeNT-treated animals occurred during the periods when the songs were most degraded, as shown on the left. **C-D** Spectral decomposition of local field potentials (LFPs) of the averaged deflections during night time at 5, 20, and 60 dpi in one control (C) and one TeNT-treated animal (D). The vertical red line depicts the trough of the raw deflection trace. The % increase is the relative increase compared to non-deflection events in the same recording timeframe of the same animal (Methods). Deflections of TeNT-treated animals in the 15–30 Hz range were approx. 27 times larger than those in control animals at 5 dpi. These differences are statistically significant between control and TeNT-treated groups, but not within the control group (p=0.7631 between controls, p<10^−35^ between all other pairs). The deflections across all animals and frequencies become more similar by 60 dpi (15–30 Hz: p=0.1371 between controls, p<10^−6^ between all other pairs; 30–70 Hz: p=0.7493 between controls, p<10^−2^ between all other pairs). For details on statistics see Methods.

We implanted a 4-shank electrode array containing 16 recording sites in both control and TeNT-injected animals. We simultaneously collected electrophysiological, head movement (recorded with the accelerometer built into the head stage), and audio recordings over a period of 90 dpi. We analyzed the spectral decomposition of the local field potentials (LFPs) during song production from an average of five vocalization epochs^12,13^. LFP recordings provide a readout of local electrical activity originating from synaptic events and action potentials^14^. In control animals, as expected, both the song behavior and LFP characteristics remained stable: the electrical activity during singing showed voltage deflections of small amplitude (Supplementary Table 1B), which were present throughout the song.

(Supplementary Figure 6 B). In contrast, at 5 dpi, TeNT-treated animals produced abnormally long vocalizations that were preceded by large voltage deflections (Supplementary table 1B), neither of which were observed in control animals (Supplementary Figure 6 A). Between 10 to 70 dpi, the songs of TeNT-treated animals were highly variable between renditions and between days. As a result of this instability in behavior, the vocalizations of perturbed animals could not be reliably aligned across trials. Hence, we focused on electrophysiological events occurring during nighttime (lights-off periods when the animals are not moving or singing), because they showed higher consistency (Figure 2 A). The neuronal activity recorded during the nighttime period will be referred to as “offline” or “lights-off” throughout this manuscript. Previous studies have hypothesized that HVC offline activity may reinforce the maintenance of adult song and could drive song learning in juveniles^15–19^. Therefore, analyzing electrophysiological activity in HVC during offline periods throughout the perturbation could reveal disruptions related to degraded behavior.

The recordings in TeNT-treated animals during lights-off offline periods contained voltage deflections that were more frequent and of larger amplitude than those of controls (Figure 2 A and B), similar to the electrophysiological differences observed between virus-treated and control animals during singing. In addition, in TeNT-treated animals, these large amplitude deflections at night occurred more frequently when the song was most degraded (Figure 2 B). At 5 dpi, these voltage deflections had an average amplitude of around −530 μV and −140 μV in TeNT-treated and control animals, respectively a 280% increase in TeNT-treated animals (Supplementary Figure 7). In TeNT-treated animals, the rate of lights-off offline voltage deflections returned to those seen in control as the song regained stereotypy around 50 dpi (Figure 2 B).

To further characterize these voltage deflections, we analyzed the LFPs (1–200 Hz) during the voltage deflection events occurring during lights-off periods, and we performed spectral decomposition of the LFPs. At 5 dpi, we observed an increase in power in the low gamma band (30–70 Hz) in TeNT-treated animals, which returned to control levels as the songs regained stereotypy (Figure 2 C and D).

The highest level of song distortion coincided with the highest rate of large amplitude voltage deflections during night-time (Figure 2 B). These findings suggest a relationship between the return to control-like offline electrophysiological activity and behavioral recovery. Previous studies mentioned the presence of some form of replay^16,17^ in HVC during sleep; here, we show that the activity during offline periods may be correlated with the reliability of song execution.

### Spontaneous local neuronal activity in HVC and RA during nighttime show opposite trends upon chronic interneuron perturbation in HVC

**Figure 3:**
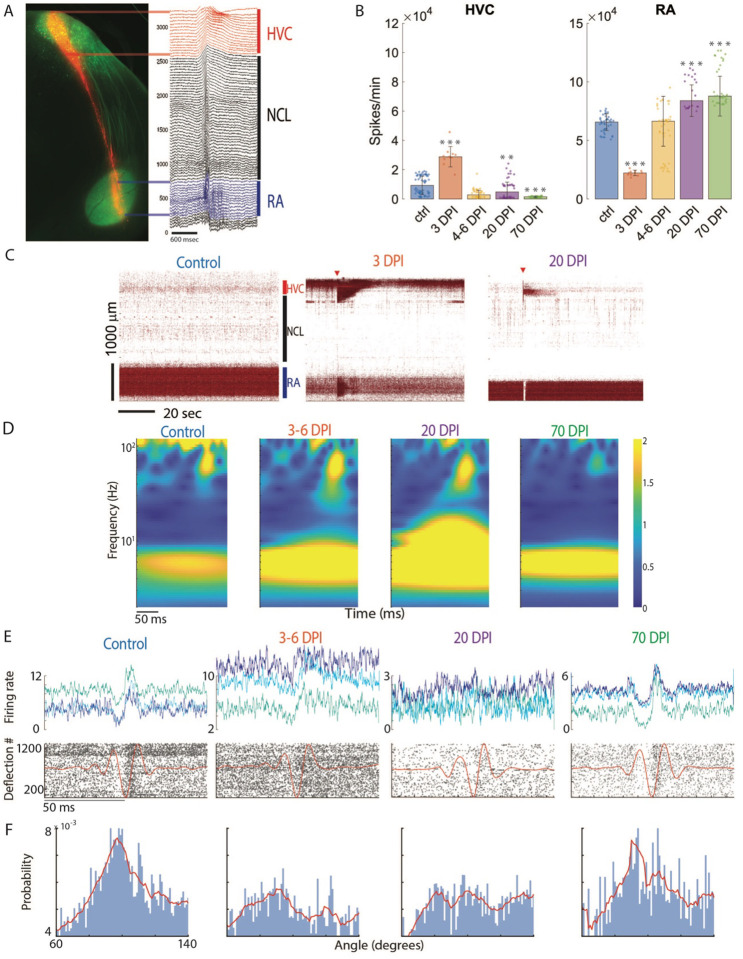
Acute electrophysiology recordings after chronic inactivation of inhibitory neurons in HVC by viral expression of TeNT. **A** A histological section example of a brain slice from a control animal shows the track of the probe (NPIX coated with DiI) insertion (red) and GFP from the viral injection (green). Note: There is green signal in RA because the control animal received injection of a virus in which GFP is driven by the ubiquitous promoter CAG in HVC. This virus infects all cell types in HVC, and axons from HVCRA neurons innervate RA. The raw electrophysiology traces are color-coded by brain area (HVC in red, NCM in black, RA in blue). **B** Average firing rates of neurons within HVC and RA in control animals (n=3) and TeNT-treated animals at 3 (n=1), 4–6 (n=4), 20 (n=4) and 70 (n=4) dpi. In HVC, the increase in average firing rate at 3 dpi after TeNT injection significantly differs from control (p-value from rank sum test: 0.4*10^−6^), as well as the decrease in average firing rate after TeNT injection at 20 dpi (p = 0.002) and 70 dpi (p = 0.57*10^−11^). In RA, the decrease in average firing rate at 3 dpi significantly differs from control (p = 0.43*10^−6^), as well as the increase in average firing rate at 20 dpi (p = 0.09*10^−9^) and 70 dpi (p = 1.45*10^−13^). The stars above the bar plots indicate statistical significance (* = p < 0.005, ** = p< 0.01, *** = p < 0.001). **C** Examples of neuronal activity in a control animal, TeNT-treated animals at 3 and 20 dpi. The red arrows highlight the “superbursts” or extreme firing levels within HVC and RA which we observed in 3 animals (two animals at 3–4 dpi and one at 20 dpi) in a total of seven instances. **D** Spectral decomposition of LFPs of the average normalized deflections during lights-off acute recordings in control animals and TeNT-treated animals at 3–6, 20, and 70 dpi. **E (top)** Example traces of the firing rate of three individual neurons during deflection events that lock or do not lock to a specific phase of the alpha oscillation (shown in red in bottom plots) in HVC of control animals and TeNT-treated animals at 3–6, 20, and 70 dpi. **E (bottom)** Examples of neuronal firing (indicated by black dots) for one neuron per condition in HVC. **F** Probability distribution of the phase of firing events relative to the alpha oscillation of the LFP in control animals and TeNT-treated animals at 3–6 dpi (n=4), 20 dpi (n=4), and 70 dpi (n=2 animals). Neuronal firing precision at specific angles (60–140) of the alpha oscillations are lost or broadened in TeNT-treated animals between 3–20 dpi and re-emerges at 70 dpi, upon song recovery.

To examine local neuronal activity at single-neuron resolution over the course of song degradation and recovery, we used high-density silicon probes (Neuropixels)^20,21^ to simultaneously record neurons in HVC, RA, and intervening brain areas (Figure 3 A). As described above, the most reliable measurements of abnormal brain activity in TeNT-treated animals were obtained during lights-off periods. Therefore, we performed Neuropixel recordings in head-fixed animals when the lights were off during their natural sleep cycle. To achieve the best possible signal-to-noise ratio, which would allow for single-neuron resolution, we focused on acute recordings that were obtained within a few hours after probe insertion. We performed acute recordings at 3–6, 20–22, and 70–77 dpi in TeNT-treated animals, and at 10 and 30 dpi in control animals to get a snapshot at that time window into both the behavior and the neuronal activity. We also included recordings from one naïve animal (not injected with any virus) to control for potential artifacts caused by the surgery or viral injection.

As a result of the loss of inhibitory tone, we observed seven events resembling seizure-like activity in HVC (n=3 TeNT-treated animals at 3–20 dpi), which propagated downstream into RA (Figure 3 C). These periods of elevated neuronal firing in TeNT-treated animals in the early days after perturbation suggest that the removal of inhibitory tone acutely leads to bursts of hyperactivity that resemble discharges previously reported during seizures^1,2,22^.

Next, we investigated overall activity levels to measure the overall HVC and RA dynamics after perturbation. In TeNT-treated animals, for the first 3–4 dpi, HVC started out with a heightened level of spontaneous nighttime activity (likely due to the loss of inhibition), but after 20 dpi, activity decreased to a level below that of control animals. Surprisingly, even after the song was fully recovered, by 70 dpi, the activity in HVC remained substantially reduced relative to control (p = 0.57*10^*−11)*^ (Figure 3 B). While activity in HVC was initially elevated and eventually decreased, RA showed the opposite trend (Figure 3 B). This observation is consistent with a prior study^23^ that showed acute changes in RA firing patterns in single neurons upon stimulation of HVC in brain slices, suggesting that HVC may directly increase the activity of RA interneurons. According to this model, an increase in overall activity in HVC (caused by blocking of HVC interneuron activity) would activate interneurons in RA, thereby reducing overall activity in RA. However, it is important to note that our recordings in RA do not allow us to identify whether the recorded activity originated from excitatory or inhibitory neurons.

### Song stereotypy recovers despite the persistence of increased power in the alpha range oscillations and decreased HVC activity during nighttime

We used acute Neuropixel recordings to analyze the neuronal firing, specifically during the offline voltage deflection events, similar to those that we initially observed using chronic recordings (Figure 2). Examples of averaged voltage traces of offline deflections recorded in the acute dataset are shown in Supplementary Figure 8. Compared to the spectral composition of the LFP signature of voltage deflections observed in the chronic recordings (Figure 2 C and D), in acute Neuropixel recordings, we detected similar changes in amplitude, power, and phase measured by the wavelet coefficient (Figure 3 D). In the LFP decomposition (continuous wavelet transform^24^) of deflection events detected in these acute recordings, we observed large differences between TeNT-treated and control animals in the alpha (1–10 Hz) and the low gamma (30–40 Hz) frequency ranges (Figure 3 D).

We calculated the difference in power from the estimated spectral density (periodogram) between the averaged (per animal) deflection and non-deflection events in the alpha (1–10 Hz) and low gamma (30–40 Hz) range. In the low gamma range (30–40 Hz), after an initial increase, the power returned to control levels at 70 dpi (Supplementary Figure 9 A). The relative phase between the alpha and low gamma oscillations during voltage deflections initially varied in angle, then returned to resemble controls by 70 dpi (Supplementary Figure 9 B–D). In contrast, the power in the alpha range stayed significantly higher in TeNT-treated animals, even after the song recovered at 70 dpi (Supplementary Figure 9 A).

Next, we compared the timing of single neuron firing with the phase of the alpha and low gamma oscillations during offline voltage deflection events. To this end, we turned to methods previously described to characterize sharp-wave ripple events during offline activity in the hippocampus^25^. We found that in control animals, the neuronal firing within HVC locked tightly to specific phases of the alpha and low gamma oscillatory events during offline voltage deflections. In TeNT-treated animals, during the song degradation period, the precision of firing with respect to low gamma oscillations in HVC or to alpha and low gamma oscillations in RA during deflection events did not change compared to control (Supplementary Figure 8). However, during the song degradation period, the precision of local neuronal firing to alpha oscillations between 60 to 140 degrees was lost or broadened compared to control and song-recovered animals (Figure 3 E and F). As song stereotypy was regained, the locking of firing to specific phases of the alpha oscillations during deflections re-emerged (Figure 3 E and F).

The finding that the neuronal firing precision locked to alpha frequency oscillations returned to control-like is unexpected, given that the power in this frequency range remained elevated even after the song recovered. Sixty days after the perturbation, the animals were able to produce a normal song despite a persistent increase in alpha power and an overall decrease and increase in spontaneous HVC and RA single-neuron activity, respectively. In sum, we found that restoration in behavioral stereotypy was paralleled by an increase in neuronal firing precision to low-frequency oscillations. However, even after the song recovered, we see long-term changes in the LFP power and offline HVC dynamics that point to large-scale restructuring in total population activity within HVC.

These observations indicate that the adult brain can overcome a permanent inactivation of a significant fraction of inhibitory neurons, which causes extended periods of abnormal activity, and precisely restore a complex behavior without recovering the original neuronal dynamics. These observations suggest that this circuit can transition into a new state capable of producing the original behavior despite the persistence of abnormal neuronal dynamics.

### Single-cell RNA sequencing reveals an increase in microglia markers and MHC class I genes during song perturbation

To investigate cellular mechanisms that might underlie the observed changes in neuronal activity and behavior at the transcriptomic level, we performed single-cell RNA sequencing (scRNAseq) of HVC from control (n=2) and TeNT-treated (n=2) adult male zebra finches at 25 dpi, around the time of peak song distortion. HVC from both hemispheres of all four birds were dissected based on retrograde tracer fluorescence and dissociated to prepare single-cell suspensions, which were indexed and pooled. This allowed the construction of a combined dataset containing results from all organisms and conditions without the need for batch correction (Supplementary Figure 11, Supplementary Table 2). Following quality control, we retained a total of 35,804 single-cell profiles spanning four individuals, consisting of two control and two TeNT-treated animals.

**Figure 4:**
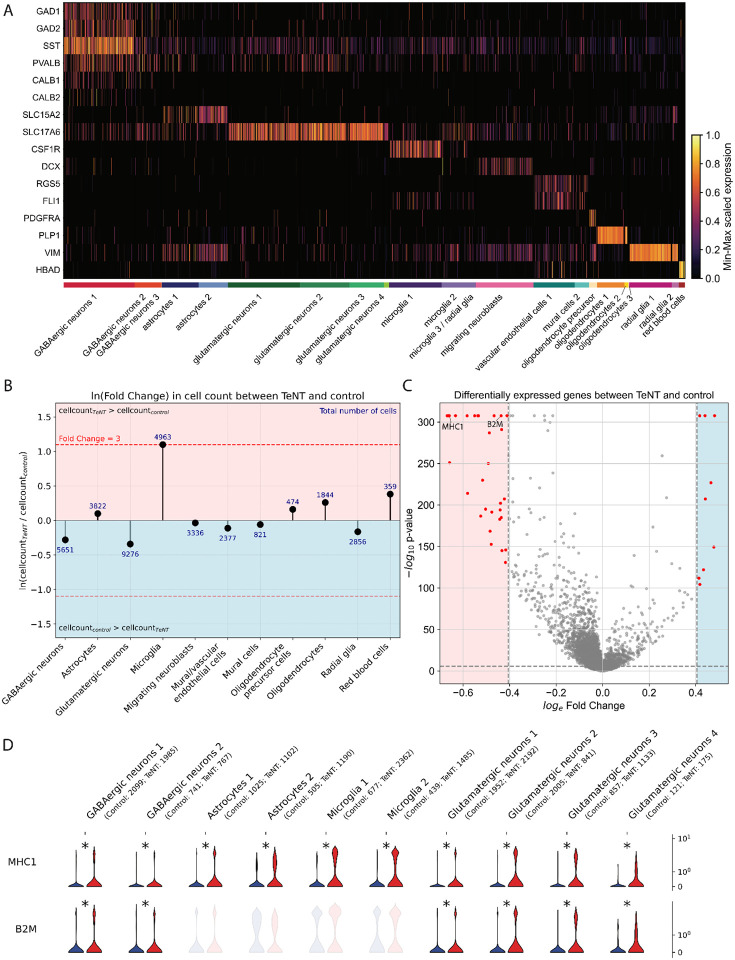
Transcriptomic changes at single-cell resolution in HVC at 25 days after chronic inactivation of inhibitory neurons through viral expression of TeNT. Single-cell RNA sequencing of the HVCs of control (n=2) and TeNT-treated (n=2) animals was performed at 25 dpi. **A** Heatmap showing min-max scaled expression of cell type marker genes for each cell type (data from both control and TeNT-treated animals). **B** Log-fold change in total number of cells per cell type between TeNT-treated and control animals. **C** Volcano plot showing statistical significance over magnitude of change of differentially expressed genes between TeNT-treated and control animals across all cell types. Dotted lines indicate fold change = 1.5 and p value = Bonferroni corrected alpha of 0.05. A list of all differentially expressed genes can be found here: https://github.com/lauraluebbert/TL_2023. **D** Violin plots of normalized counts of major histocompatibility complex 1 α chain-like (MHC1) (ENSTGUG00000017273.2) and beta 2 microglobulin-like (B2M) (ENSTGUG00000004607.2) genes in control (n=2, blue) and TeNT-treated (n=2, red) animals per cell cluster. A star indicates a significant increase in gene expression in TeNT-treated animals compared to control (p < 0.05 and fold change > 1.5).

While cell type abundance was highly concordant between replicates of the same condition (Supplementary Figure 11, Figure 4 A), we found that animals treated with TeNT displayed a three-fold increase in the number of microglia (Figure 4 B). It is unlikely that this increase in microglia was due to an inflammatory reaction caused by the surgical procedure or the AAV injection because control animals also received a viral injection with an identical viral backbone that only expressed a fluorescent protein (NeonGreen), which did not affect neuronal activity. Thus, we hypothesize that the increase in microglia in TeNT-treated animals is a consequence of the chronic muting of inhibitory neurons.

Several studies have shown that microglia play a role in synaptic plasticity during early brain development and learning in mammals^26–29^. These prior observations, in combination with our findings, suggest that microglia might participate in some form of synaptic reorganization triggered by the perturbation of neuronal activity caused by the permanent muting of interneurons. Consistent with this hypothesis, previous epilepsy studies found upregulation of microglia associated with neuronal reorganization following seizure-like activity in mammalian circuits^30,31^. To validate the scRNAseq findings, we performed *in situ* hybridization (ISH) using a probe against RGS10, a gene expressed in microglia, during song degradation at 25 dpi and after recovery at 90 dpi. At 25 dpi, the number of microglia increased in TeNT-treated animals compared to control (Supplementary Figure 12 A, 13 A and B), and returned to control levels by 90 dpi, when the song had recovered.

To further investigate the hypothesis that microglia may be associated with circuit reorganization involved in neuronal plasticity, we counted the number of microglial cells in HVC at different times during song learning in naive (untreated), juvenile birds using ISH. The number of microglia in the HVC of juveniles was higher during the early stages of the song learning period (30–50 days post-hatching (dph)), compared to 70 dph, after the song became more stereotypic (Supplementary Figure 12 B, 13 C and D).

scRNAseq analysis also revealed a significant increase in the expression of the α chain of major histocompatibility complex class I (MHC I) and β_2_-microglobulin (B2M) genes across several neuronal cell types in TeNT-treated animals (Figure 4 C and D). We confirmed the increases in MHC I by ISH (Supplementary Figure 12 C, 13 E and F). MHC class I molecules are heterodimers that consist of two polypeptide chains, α and B2M, and are involved in antigen presentation for T cells^32^. This increase in MHC I and B2M is likely triggered by the genetic silencing of inhibitory neurons, and not due to an inflammatory response because it was not observed in control animals injected with the control virus that just expressed a fluorescent protein. MHC I and B2M have been observed in previous studies to be highly expressed in neurons during brain development^33,34^, consistent with a hypothetical role in synaptic plasticity^35,36^. Moreover, a human study of epilepsy observed an increase in MHC I in neurons and glial cells^37^. Here we observe that MHC I and B2M are upregulated in response to perturbation of neuronal activity in a fully formed circuit, suggesting their possible role in the restoration of brain function after chronic loss of inhibitory tone.

### Permanent genetic muting of inhibitory neurons in HVC leads to zebra finch song degradation and recovery that does not require a feedback circuit essential for song learning

The increases in microglia observed both in juvenile animals while they are learning the song, and in adult animals after HVC perturbation suggests the possibility that some of the mechanisms mediating song recovery in adults and song learning in young animals may be shared. To test this hypothesis, we set out to test if adult song recovery after manipulation of interneurons required the same feedback circuit that is essential for song learning.

Juvenile animals start singing a highly variable and unstructured song, and by continuous trial and error over 2–3 months and thousands of practice renditions their song “crystallizes” into a stable motif with minimal variability^38,39^. Song learning in juveniles requires the lateral portion of the magnocellular nucleus of the anterior neostriatum (LMAN), another nucleus in the song system that provides input to RA, the downstream target of HVC^40–42^. During learning, LMAN injects noise into RA to enable the exploration of different states^43^ and gates the plasticity of synapses from HVC to RA^44^. In addition to its role in learning, LMAN is also necessary for song modification in adult animals^45^. Deafening adult animals causes a progressive degradation of their songs over several weeks, which can be blocked by prior LMAN lesioning. Similarly, a previous study demonstrated that small, focal electrical lesions in HVC in adult animals caused their songs to degrade, and that the song degradation could be prevented by lesioning LMAN^46^. Therefore, it is possible that when HVC is perturbed in adult animals, the ‘noisy’ input from LMAN to RA becomes more dominant than the input from HVC, and this causes song degradation. To test this hypothesis, we first chemically lesioned LMAN by bilateral ibotenic acid injection, and 10 days later we injected AAV-dlx-TeNT into HVC (n=3). We observed that in animals with AAV-dlx-TeNT injection into HVC of animals with a prior LMAN lesion, the song degraded and recovered in the same time-scale as animals with intact LMAN, and in a similar way as in animals with intact LMAN, indicating that song degradation after HVC perturbation is not caused by an exacerbated LMAN influence on the song system (Supplementary Figure 3–5). Our finding that animals degraded and recovered their song without LMAN indicates that LMAN is not required for the song to recover after perturbing the activity of HVC interneurons in adult animals. This is surprising given that spontaneous RA offline activity exhibits large changes during the perturbation and recovery process, which, based on previous work^47,48^, would be hypothesized to require some re-tuning involving LMAN. These findings indicate that the recovery following perturbation of activity in HVC in adults likely occurs by different mechanisms than song learning in juveniles, which requires the LMAN nucleus^41,49^.

## Discussion

The brain requires a balance between excitation and inhibition to maintain physiological activity patterns and enable reliable behaviors. We found that permanently muting a large fraction of inhibitory neurons in a pre-motor circuit in adult songbirds severely disrupts song, a complex, learned behavior for an extended period of time (30–90 days). However, despite this long-term disruption, animals can eventually recover and produce a song highly similar to the original.

We investigated the electrophysiological and transcriptomic changes induced by this perturbation to understand the cellular mechanisms responsible for this behavioral recovery. First, we detected changes in voltage deflection events as a readout of offline dynamics (during the lights-off period) within HVC. We observed an increase in the rate and amplitude of these deflection events while the song was degraded, which returned to control-like once the song regained stereotypy. To gain a fine-scale understanding of these offline dynamics, we investigated single and multi-neuronal activity within HVC and in its downstream nucleus RA. After muting interneurons in HVC, we observed two changes in HVC dynamics that remained abnormal even after the song had fully recovered: a decrease in offline single neuron firing and an increase in alpha frequency power. It has been hypothesized that alpha-frequency LFP signals could be caused by synaptic inputs into a brain region. In this case, the decrease in neuronal firing in HVC that we observed may have triggered a compensatory increase in the synaptic inputs from upstream nuclei projecting into HVC (such as Uva and Nif), which could account for the observed increase in alpha power. Alternatively, this increase in alpha power could originate from synaptic inputs from neurons within HVC. Future experiments will be necessary to understand how the perturbations of HVC affect the dynamics of other nuclei in the song system.

To investigate the relationship between HVC oscillation and local neuronal dynamics, we measured the precision of single neuronal firing to specific phases of alpha and gamma oscillations, where we observed significant changes upon perturbation. In control animals, single neuronal activity within HVC showed precise firing to certain phases of alpha and gamma oscillations during offline voltage deflections. In contrast, after interneuron muting, during the peak song distortion, single neuronal firing did not align to specific phases of alpha frequency oscillations. Then, as the song recovered, the precision of firing to the phase of alpha oscillations returned to normal.

The relationship between single neuronal firing and alpha oscillations in the song system is not understood, but it could represent how the local HVC circuit responds to inputs from other brain areas. One hypothesis consistent with our observations is that during song degradation when stereotypy of the behavior is lost, the local HVC circuit is unable to properly respond to synaptic inputs, which could result in the observed desynchronization of spikes to alpha oscillations. As the song comes back to normal, the precision of local neuronal firing recovers even though the power stays permanently elevated in the alpha range. These findings suggest that the motor circuit is able to execute behavior properly even in the face of permanent changes to its properties, such as increased alpha frequency power and low spontaneous offline neuronal activity in HVC.

Next, we investigated the transcriptomic changes during song degradation and recovery. We found a 3-fold increase in microglial marker genes in our sc-RNAsequencing data compared with control animals. This increase was confirmed via *in situ* hybridization and shown to return to control levels as the song recovered. Microglia are known for playing a role in synaptic changes during learning and circuit establishment^26,28,29^. Moreover, several works have shown an increase in microglia after seizures in rodents and humans^27,30,31^. Our study suggests that microglia may also be involved in the restoration of brain function. Moreover, we found an increase in microglia during the early stages of song learning in juvenile animals by in situ hybridization.

Our experiments suggested that the song recovery in adults following HVC perturbation has some superficial similarities (variable song production and increase in microglia numbers) to the process by which juveniles learn their songs. However, we found that there is a fundamental difference between these two processes. In juveniles, song learning requires that LMAN provides input to RA, and it has been hypothesized that LMAN injects noise into RA such that inputs from HVC into RA can explore different states^40,50^. Moreover, previous works indicate that LMAN is also required to enable song changes even in adults^48,51^. In contrast, here we found that recovery after chronic genetic muting of inhibition in HVC can occur without LMAN. These observations reveal that the adult brain’s mechanisms to recover behavior after disruptions in neuronal activity differ from those used to acquire the behavior as juveniles.

We wondered if the mechanisms of behavioral recovery in an adult are similar and whether the activity of excitatory or inhibitory neurons is selectively perturbed. In a previous publication, we observed that permanent genetic muting with TeTN of a large fraction of projection neurons in HVC led to a degraded song, which recovered in around 2 weeks^52^. In this work, we observe that permanent genetic muting of a large fraction of interneurons also leads to degradation and recovery of the song, albeit the recovery takes around 2 months in this case. Taken together, our previous work and this study show that HVC is able to restore functional dynamics after two “orthogonal” perturbations to the circuit, highlighting the resilience of this nucleus. Our results indicate that HVC can still precisely execute a learned song, a complex behavior, despite a significant reduction in the fraction of active inhibitory neurons and an overall abnormal activity in HVC.

These findings suggest that the successful functional recovery of behavior after a brain perturbation can occur without the restoration of the normal dynamics of the circuit. Instead, the circuit seems to adapt and reorganize into a new state capable of producing the original behavior. This adaptive process may be crucial for maintaining behavioral accuracy over long periods of time and could represent a key feature of brain resilience.

## Methods

### Animals

The Institutional Animal Care and Use Committee of the California Institute of Technology approved all procedures involving zebra finches. All birds used in the current study were bred in our own colony and housed with multiple conspecific cage mates of mixed genders and ages until used for experiments. Before any experiments, adult male birds (>120 days post-hatching (dph)) were singly housed in sound isolation cages with a 14/10 hr light/dark cycle for >5 days until they habituated to the new environment and started singing. Thereafter, birds were kept in isolation until the end of the experiment.

### Behavioral recordings

Adult male zebra finches’ (n=30, 130–890 dph) undirected songs were recorded 24/7 in sound-isolated chambers for 10–14 days before any manipulation to get a baseline of their song. Recordings were done with microphones (Audio-technica, AT831b) that are connected to an amplifier M-TRACK 8 and recording software Sound Analysis Pro 2011 at 44100 Hz. Animals were housed in these chambers and continuously recorded for the duration of the experiments.

### Viral vectors

AAV-TeNT contained the promoter from the human dlx5 gene driving expression of the light chain of tetanus toxin fused to EGFP with a PEST domain. AAV9-dlx-TeNT was obtained from the Duke viral core facility. The control virus used was AAV9-CAG-NeonGreen, where CAG drives the expression of NeonGreen, which is a GFP variant.

### Stereotaxic injection

Birds were anesthetized with isoflurane (0.5% for initial induction, 0.2% for maintenance) and head-fixed on a stereotaxic apparatus. First, to inject a retrograde tracer in area X, craniotomies were made bilaterally and fluorescent tracers (fluoro-ruby 10%, 100–300 nL) were injected through a glass capillary (tip size ~25 μm) into the corresponding nuclei (coordinates from dorsal sinus in mm - area X: Anteroposterior (AP) 3.3–4.2, Mediolateral (ML) 1.5–1.6, Deep (D): 3.5–3.8). To deliver the virus (AAV) into HVC, a second surgery was performed 7–10 days after retrograde tracer injection. By then, HVC was strongly labeled by fluorescence and visible through a fluorescent stereoscope. AAVs diffuse extensively (~500 μm), and a single injection (~100 nL) in the center of HVC was sufficient to label enough cells. All injections in HVC were performed at ~20 nL/min to minimize physical damage. At the end of every surgery, craniotomies were covered with Kwik-Sil, and the skin incision was closed with Gluture.

#### LMAN lesion

We used ibotenic acid (2%) dissolved in 100mM NaOH to chemically ablate LMAN, in adult male zebra finches (n=4) between 120–200 dph. Coordinates used with 50 degree head angle were (3.2/3.5/3.8 mm AP; 1.7/1.8/2 mm ML ; 2.4;2.8/2.4;2.7/2.3;2.5 mm Deep). We achieved ablation of ~80–98% of LMAN bilaterally confirmed by CGRP staining. We initially measured the acoustic variability within each syllable before and after lesion and found that it decreased in 3/4 animals (which agrees with previous findings) to confirm the success of our LMAN lesions. Next, 7–10 days following the LMAN ablation we followed the previously describe stereotaxic injection protocol for tracer and virus injection. The one animal that did not have decrease in syllable level acoustic variability following lesion later on by histology was also confirmed to only receive a partial lesion therefore we removed this animal from further behavioral analysis.

### Chronic electrophysiology recordings

Animals (n=4, 300–700 dph) were implanted in the right hemisphere HVC with 4 by 4 electrode arrays (Neuronexus A4×4–3mm-50/100-125-703-CM16LP) based on retrograde fluorescent labeling of HVC (just as for viral injections). Post-perfusion histology images were obtained to locate the electrode array within HVC for each animal (Supplementary Figure 15). Electrode implantation occurred within the same surgery as the viral injection.

This procedure follows the same surgical steps as the viral delivery protocol, until the point of electrode implantation. A small opening was cut on the dura (just big enough to fit the electrode array) to lower the electrodes manually. The reference and ground were a gold screw pin placed into the cerebellum. The skin was removed from the surface of the skull for the majority of the surface, in order to secure the implant. Before implantation, the skull and the craniotomies were cleaned with saline and dried and the skull was prepared according to the protocol of the C&B Metabond cement system. Post implantation we covered the craniotomies with kwik-sil. Once hardened, we covered the whole skull, and the part of the electrode still exposed, with metabond. The head stage (Intan RHD Part # C3335) was connected to the probe before implantation and securely metabonded to the connection between the probe and head stage in order to prevent detachment when the bird is moving. SPI interface cables (Intan Part #C3203, #C3213) were connected to the acquisition board (Open Ephys). Data was recorded at 30,000 Hz with the Open Ephys software system. Animals were freely moving with a passive counterweight-based commitator system.

### Acute electrophysical recordings

Animals (n=10, 140–250 dph) went through the same surgical procedure as described for a stereotaxic viral injection. However, at the end of the surgery the skin was removed from the skull, and the whole skull was pre-treated and covered in metabond except for the craniotomies over HVC that were covered with kwik-cast until the day of the acute recording session. Shortly before the recording session, a head-bar was glued on top of the frontal surface of the metabonded skull to allow the head-fixation of the bird for the recording session. Then, the kwik-cast was removed from the craniotomy over HVC (left or right hemisphere or both depending on the animal) and a small incision was made in the dure over HVC, which was identified by the retrograde tracer previously injected. The ground was placed into the cerebellum. Then the high-density silicone probe (Neuropixel) was lowered with a motorized arm over hours for 2.6–3 mm deep into the brain. The head stage and acquisition board was connected to the computer and data was recorded with the Open Ephys software. Once the probe settled in the brain, we had 4 distinct recording sessions. Post-perfusion histology images were obtained to locate electrode array within HVC for each animal (Supplementary Figure 16). Recording sessions: lights on silence (10 min), followed by playback of the bird’s own song (3–10 min); lights-off silence (10 min), followed by playback of the bird’s own song (3–10 min); microinjection of 100 nL 250 μM Gabazine (Hellobio, HB0901), followed by the same protocol of lights-off and on without Gabazine.

### Song analysis

Song analysis was performed using MATLAB (MathWorks).

#### Song feature parameterization

Continuous audio recordings (44.1 kHz) were segmented into individual bouts manually. We used the open-source Matlab software package, Sound Analysis Pro 201^53^ to generate spectrograms and derive non-linear, time-varying song parameterizations. The time-varying features were: pitch, goodness of pitch, Wiener entropy, log-power of the spectrogram, mean frequency, and the maximum power in each quadrant of the frequency range 0– 11 kHz (labeled power 1, power 2, power 3, and power 4). These features were computed across the entire bout every 1 ms. These time-varying features were the base from which various portions of the song were further characterized.

#### Syllable parameterization

To quantify the change in the acoustic structure of the song, we tracked song features over the course of the perturbation. In Fig 1, we took the average feature values over all song segments in the three closest days of recorded song for each day plotted. We extracted the average mean frequency, average pitch, maximum goodness of pitch, maximum log power, minimum entropy, and entropy variance over the first 50 ms of each song segment, or the entire song segment if the segment was less than 50 ms. We centered and normalized feature values by the mean and standard deviation of the distribution of feature values in syllables five days pre-perturbation to compare trends across birds.

To visualize how the population of song segments evolved relative to the original syllable structure (Fig 1B), we plotted song segments as points in the first 2 principal components of the 7-dimensional acoustic feature space composed of: average mean frequency, average pitch, maximum goodness of pitch, maximum log power, minimum entropy, and entropy variance over the first 50 ms of each song segment, and the full duration of each song segment. 500 syllables recorded pre-perturbation were labeled by hand, and contour plots were made at the mean of each labeled stereotyped syllable type for visual reference as the song degrades and recovers. Principal component dimensions were computed on the 500 labeled syllables, and subsequent song segments were projected into this principal component space.

#### Syllable segmentation

We identified syllables and silences within each bout by imposing thresholds on the time-varying, total log-power of the spectrogram. We selected the threshold based on manual inspection for each bird. We then performed a smoothing step wherein periods of silence less than 15 ms were converted to song segments. Song segments less than 20 ms were excluded from the analysis. Segmentation was further checked by eye by random sampling across both stereotyped motifs and degraded song. We then applied the power threshold to the entire course of song recordings for each bird.

A note on terminology: we refer to song segments to indicate continuous periods of singing. In the unperturbed song, these song segments are termed syllables. Because this is a continuous recovery process, these terms sometimes overlap in our usage.

#### Syllable timing distributions

Syllable durations were extracted from the syllable segmentation process. Discrete distributions of syllable durations were computed by normalizing each distribution of all song segment durations within individual days such that the sum of each daily distribution over all binned durations equaled one. Distributions for individual days were then assembled into a matrix wherein the columns represented normalized distributions for individual days. This matrix was plotted as a heat map in Supplementary Figure 1.

#### Continuous representation of bout trajectory

We generated continuous visualizations of bouts across the entire perturbation trajectory as shown in the graphical abstract^54,55^. We randomly sampled 100 bouts from single days of recording to build a representative sample of the song over the course of the experiment. For each bout, we slid a 150 ms window in 3 ms steps along the bout length. We then generated a high-dimensional, acoustic parameterization of each 150 ms song window by taking the moving average in 20 ms segments every 5 ms of six song features (mean frequency, pitch, goodness of pitch, power 4, power 2, and summed log power of the spectrogram). We performed principal component analysis on this high-dimensional set across all bouts to reduce the feature set to 30 dimensions.

### Analysis of the chronic electrophysiology recordings

#### Preprocessing of Data

Bad channels were removed from analysis by visual inspection of the first minute of each recording. Three of the four birds studied had one channel that had behavior that was an order of magnitudes larger than all others and data from this channel was removed for further analysis.

#### Detection of sharp waves

Accelerometer and electrophysiology data were resampled to 125 Hz from the original 30,000 Hz using the Matlab resample function. Since accelerometer data was not zero-centered, the mean was subtracted before resampling and then added back to avoid edge effects from the lowpass filtering during resampling. Sharp waves were detected from the downsampled data using a procedure adapted from a zebra finch sleep study^56^ and were only detected during periods of low movement. This was determined by setting a threshold on the moving standard deviation of the velocity as movement events appeared as large fluctuations in the velocity data. Briefly, the local field potential (LFP) traces were averaged across all electrodes and bandpassed from 1 to 40 Hz using MATLAB’s bandpass function. Putative events were detected by peaks in the inverted (−1x) trace and were in the lowest 5% of all values during non-movement, were at least 10 ms long, and were 80 ms apart. These putative events were used to construct a template by taking the average 80 ms trace centered on each event. Cross-correlating this template with the entire overnight LFP trace and taking the z- score (mean and SD from only non-movement times) revealed events that closely matched the template. Peaks were detected in the z-scored cross-correlation and defined as events if they were above a given threshold and were at least 80 ms apart. Notably, thresholds were not tuned across birds or recordings but a constant threshold was sufficient in all birds to detect events. Extraction of sharp waves. The detected events were used to index the original 30,000 Hz data. 500 ms of data was extracted for each event and recentered on the minimal value in the center 100 ms. An equal number of 500 ms non-sharp wave events were extracted from a random selection of times without movement or sharp waves. Sharp waves with clear electrical noise were omitted from further analysis.

#### Multi-unit activity (MUA)

MUA was detected using an established method^57^. Briefly, channels were common averaged referenced, bandpassed from 600 to 6,000 Hz, and upsampled to 50,000 Hz. The square root of the local energy operator (Mukhopadhyay & Ray, 1998) was taken and then Gaussian smoothed (σ = 0.4). Thresholding this trace and then downsampling back to 30,000 Hz gave the times of high MUA.

#### Time-frequency analysis

The continuous wavelet transform was used to compute the spectra for each sharp wave. Percent increase relative to mean power spectra were averaged within each day and bird. The spectrum of average power in the center 33 ms of each event was used to compute the percent increase in power relative to non-sharp wave events at each frequency. To determine the presence of phase-amplitude coupling between low-frequency (10–20 Hz) and gamma-frequency (30–40Hz) bursts, the phase of the low-frequency band, and power of the high-frequency band were extracted using the Hilbert transform of the band-passed signal. Determining whether the peak gamma activity occurred at a consistent phase of the low-frequency oscillation, could suggest phase-amplitude coupling. To further assess this, we asked at what time and frequency a max in the spectrum occurred for each event, relative to the minima of each sharp wave. We also wanted to ask whether the power of deflections in different frequency bands differed significantly across control and experimental birds. We computed the relative increase in power in the frequency bands of interest (15–30 Hz, and 30–70 Hz) during a deflection relative to non-deflection times. We then used a t-test to statistically test whether power differed significantly across birds for a given day.

### Analysis of the acute electrophysiology recordings

The low-frequency electrophysiology data was recorded at 2,500 Hz while the high-frequency data was recorded at 30,000 Hz. A MATLAB script was written to analyze the low- and high-frequency oscillations. All low-frequency data was high pass filtered (1–300 Hz) and median subtracted. All high-frequency data was common average referenced and low-pass filtered (300–7,000 Hz). For deflection event detection, we used two methods. One to use a template per animal for extraction of deflection and non-deflection events with template matching in a semi-automated manner. Second, manually picked out peaks that were 9 standard deviations away from the average raw signal, then further manually curated these events. Most of our further analysis was done using the deflection events that resulted from template matching (except Supplementary Figure 10A for the difference in power between deflection and non-deflection event calculation). For spike detection, we used two separate methods, one to threshold the high-frequency data to only consider spikes that are 9 standard deviations above the average signal in a 2-minute window. The second method used for spike detection was using the Kilosort software v2.5^58^ to extract spikes from the raw data. Next, we manually curated the dataset to capture the waveform and firing rate of neurons. The thresholded spiking data was used to generate Figure 3BC. We performed statistics (Wilcoxon, rank sum test) to show statistically significant firing rates related to control in HVC and RA in Figure 3B.

To calculate the power difference between deflection and non-deflection events in the acute recording in Supplementary Figure 11 A, we used a script developed for the chronic recordings with the use of the built-in MATLAB function *pwelch*. To analyze the spectral decomposition of the LFP signature (Figure 3D) in control and TeNT-treated animals during the experimental timeline we calculated the Morse continuous wavelet transform using the built-in MATLAB function. From this finding, we obtained the mean oscillation at the alpha range (1–10 Hz) and at low gamma (30–70 Hz). Then, we performed an iterative alignment process of these two oscillations and calculated the angle of the low-frequency oscillation (from obtaining the Hilbert transform) at the peak amplitude of the high-frequency oscillation (Supplementary Figure 11BC). Then we proceeded to align the single neuronal firing (obtained from Kilosort) to the alpha and the low gamma oscillations and plotted the normalized probability distribution (Figure 3F) of neurons firing at a specific phase (angle) of the alpha or low gamma oscillations. We performed the Kolgomorov-Smirnov test on the cumulative density function (CDF) (Supplementary Figure 11D) to assess if the change in probability distribution in the relationship between alpha and low gamma oscillations is statistically significant from control distributions at 3–6, 20, and 70 dpi.

### Single-cell RNA sequencing

#### Animals

All of the work described in this study was approved by California Institute of Technology and Oregon Health & Science University’s Institutional Animal Care and Use Committee and is in accordance with NIH guidelines. Zebra finches (Taeniopygia guttata) were obtained from our own breeding colony or purchased from local breeders.

#### Dissociation and cDNA generation

Animals were anesthetized with a mix of ketamine-xylazine (0.02 mL / 1 gram) and quickly decapitated, then the brain was placed into a carbogenated (95% O2, 5% CO2) NMDG-ACSF petri dish on ice. The brains were dissected on a petri dish with NMDG-ACSF surrounded by ice under an epifluorescent microscope guided by the fluoro-ruby retrograde tracing from Area X to HVC.

We used the commercially available Worthington Papain Dissociation system with some minor changes and add-on steps. We followed all the steps included in the Worthington protocol with a final concentration of 50 U/mL of papain. To match the intrinsic osmolarity of neurons in zebra finches we used NMDG-ACSF (~310 mOsm) instead of the EBSS for post-dissection and STOP solution. Another modification was to add 20 μL of 1 mg/mL Actinomycin D (personal communication from Allan-Hermann Pool;^59^) into 1 mL of the post-dissection medium and the STOP solution in which trituration occurred. Papain digestion occurred for an hour on a rocking surface with constant carbogenation in a secondary container above the sample vial at RT. We performed trituration with increasingly smaller diameter glass pasteur pipettes. Trituation was performed inside the papain solution. Then, once the tissue was fully dissociated, we centrifuged the samples at 300 g RT for 5 minutes and resuspended them in STOP solution. Next, we used a 40 μm Falcon cell strainer pre-wet with the STOP solution and centrifuged again at 300 g RT for 5 min. Finally, we resuspended the cell pellet in 60μl of STOP solution and proceeded to barcoding and cDNA synthesis. The cell barcoding, cDNA synthesis, and library generation protocol were performed according to the Chromium v3.1 next GEM single cell 3’ reagent kits by Jeff Park in the Caltech sequencing facility. Sequencing was performed on an Illumina Novaseq S4 sequencer with 2×150 bp reads.

#### Generation of count matrices

The reference genome GCA_003957565.2 (Black17, no W) was retrieved from Ensembl on March 20, 2021 (http://ftp.ensembl.org/pub/release-104/gtf/taeniopygia_guttata/). We quantified the gene expression in each of the four datasets using the kallisto-bustools workflow^60^. The reference index was built using the kb-python (v0.26.3) *ref* command and the above-mentioned reference genome. Subsequently, the WRE sequence was manually added to the cdna and t2g files generated by kallisto-bustools to allow the identification of transgenic cells. The count matrix was generated for each dataset using the kallisto-bustools *count* function. The resulting count matrices were compared to those generated by the 10X Cell Ranger pipeline (v6.0.1) and kallisto-bustools *count* with *multimapping* function. For all four datasets, kallisto-bustools mapped approximately 10% more reads than Cell Ranger (Supplementary Figure 17). No increase in confidently mapped reads was observed when using the multimapping function, indicating that reads align confidently to one gene in the reference genome (Supplementary Figure 17).

#### Quality control and filtering

The datasets were filtered separately based on the expected number of cells and their corresponding minimum number of UMI counts (Supplementary Figure 11). Following quality control based on apoptosis markers and library saturation plots (Supplementary Figure 11), the count matrices were concatenated and normalized using log(CP10k + 1) for downstream dimensionality reduction and visualization using Scanpy’s (v1.9.1;^61^) *normalize_total* with target sum 10,000 and *log1p*. Gene names and descriptions for Ensembl IDs without annotations were obtained using gget (v0.27.3;^62^).

#### Dimensionality reduction and normalization

The concatenated data was mapped to a lower dimensional space by PCA applied to the log-normalized counts filtered for highly variable genes using Scanpy’s *highly_variable_genes*. Next, we computed nearest neighbors and conducted Leiden clustering^63^ using Scanpy.

Initially, this approach was performed on the control and TeNT datasets separately. This resulted in the identification of 19 clusters in the control data and 22 clusters in the TeNT data (Supplementary Figure 11). For both conditions, equal contribution from both datasets indicated that there was minimal batch effect, as expected since the data was sequenced in a pooled sequencing run. We also performed batch correction using scVI^64^ which did not change the contribution of each dataset per cluster. As a result, we continued the analysis using the data that was not batch-corrected with scVI.

Next, we concatenated all four datasets and followed the approach described above. This resulted in the identification of 21 Leiden clusters, which we also refer to as cell types (Figure 4 A). Each cluster was manually annotated with a cell type based on the expression of previously established marker genes^65^.

The cell type annotation was validated by the top 20 differentially expressed genes extracted from each cluster using Scanpy’s rank_genes_groups (P values were computed using a t-test and adjusted with the Bonferroni method for multiple testing) (Supplementary Figure 12). Clusters identified as glutamatergic neurons were further broken down into HVC-X- and HVC-RA-projecting glutamatergic neurons using previously established marker genes (data not shown; also see https://github.com/lauraluebbert/TL_2023). We found that reclustering all cells labeled as glutamatergic neurons using the Leiden algorithm did not yield different results and we therefore continued with the initial clusters (data not shown). All results discussed in this paper were confirmed by both jointly and separately clustering the experimental conditions.

#### Comparative analysis of clusters and conditions

Differentially expressed genes between clusters were identified using Scanpy’s *rank_genes_groups* (p values were computed using a t-test and adjusted with the Bonferroni method for multiple testing, and confirmed by comparison to P values generated with Wilcoxon test with Bonferroni correction). In the violin plots, unless otherwise indicated, a star indicates a p value < 0.05 and a fold change > 1.5 difference in mean gene expression between the indicated conditions (p value computed with scipy.stats’ (v1.7.0) *ttest_ind* and adjusted with the Bonferroni method for multiple testing).

### *In situ* hybridization

#### Animals

All of the work described in this study was approved by the California Institute of Technology and Oregon Health & Science University’s Institutional Animal Care and Use Committee and is in accordance with NIH guidelines. Zebra finches (Taeniopygia guttata) were obtained from our own breeding colony or purchased from local breeders. Developmental gene expression in HVC in the 20-, 50-, and 75-days post-hatch (dph) male and female zebra finches was assessed as previously described^66^. The sex of birds was determined by plumage and gonadal inspection. Birds were sacrificed by decapitation, bisected in the sagittal plane and flash-frozen in Tissue-Tek OCT (Sakura-Finetek), and frozen in a dry ice/isopropyl alcohol slurry. Brains of TeNT-manipulated finches were coronally blocked anterior to the tectum and flash frozen in Tissue-Tek (Sakura). All brains were sectioned at 10um on a cryostat and mounted onto charged slides (Superfrost Plus, Fisher).

#### In situ hybridization

*In situ* hybridization was performed as previously described^67,68^. Briefly, DIG-labeled riboprobes were synthesized from cDNA clones for RGS10 (CK312091) and LOC100231469 (class I histocompatibility antigen, F10 alpha chain; DV951963). Slides containing the core of HVC were hybridized overnight at 65°C. Following high stringency washes, sections were blocked for 30 min and then incubated in an alkaline phosphatase conjugated anti-DIG antibody (1:600, Roche). Slides were then washed and developed overnight in BCIP/NBT chromogen (Perkin Elmer). To minimize experimental confounds between animals, sections for each gene were fixed together in 3% paraformaldehyde, hybridized with the same batch of probe, and incubated in chromogen for the same duration.

Sections were imaged under consistent conditions on a Nikon E600 microscope with a Lumina HR camera and imported into ImageJ for analysis. We quantified the expression level of the gene as measured by optical density and the number of cells expressing the gene per unit area, as previously described^66^.

Optical density was measured by taking the average pixel intensity of a 300×300 pixel square placed over the center of HVC. This value was normalized to the average background level of the tissue. To quantify the number of labeled cells, we established a threshold of expression that was 2.5x the background level. Binary filters (Close-, Open) were applied and the number of particles in the same 300×300 pixel square was quantified.

### Histology

After cardiac perfusion with room temperature 3.2% PFA in 1xPBS we let the brains fix for 2–4 hours at room temperature. After each hemisphere of the brain was sectioned sagittally with a vibratome at 70–100 μm thickness. The brain slices containing HVC were collected and incubated at 4 C overnight with the primary rabbit anti-GFP (AB3080P, EMD Milipore) (blocked in 10% donkey serum in 0.2% Triton 1xPBS). On the second day, the brains were washed in 0.05% Triton 1xPBS and incubated for 2 hours in the dark at room temperature in the secondary goat anti-rabbit 488 (ab150077). Next, the brain slices were washed and mounted in Fluoromount (Sigma). Confocal images were taken with the LSM800.

## Supplementary Material

1

## Data Availability

Data generated in this study have been deposited in Caltech DATA and can be found at the following DOIs: https://doi.org/10.22002/ednra-nn006 and https://doi.org/10.22002/3ta8v-gj982. Please do not hesitate to contact the authors for data or code requests. The code used for the analysis of the single-cell RNA sequencing data can be found here: https://github.com/lauraluebbert/TL_2023. The code used for the analysis of the chronic electrophysiology data can be found here: https://github.com/jordan-feldman/Torok2023-ephys.
