# Supplementary material for: Recovery of a learned behavior despite partial restoration of neuronal dynamics after chronic inactivation of inhibitory neurons
Source: bioRxiv. 2023 Dec 15:2023.05.17.541057. Preprint. [Version 6] doi: 10.1101/2023.05.17.541057 (PMC10245685; doi:10.1101/2023.05.17.541057)
Supplement: 1 [file NIHPP2023.05.17.541057V6-supplement-1.pdf]

bioRxiv preprint doi: https://doi.org/10.1101/2023.05.17.541057; this version posted December 15, 2023. The copyright holder for this preprint (which was not certified by peer review) is the author/funder, who has granted bioRxiv a license to display the preprint in perpetuity. It is made available under aCC-BY 4.0 International license.

Supplementary Figures

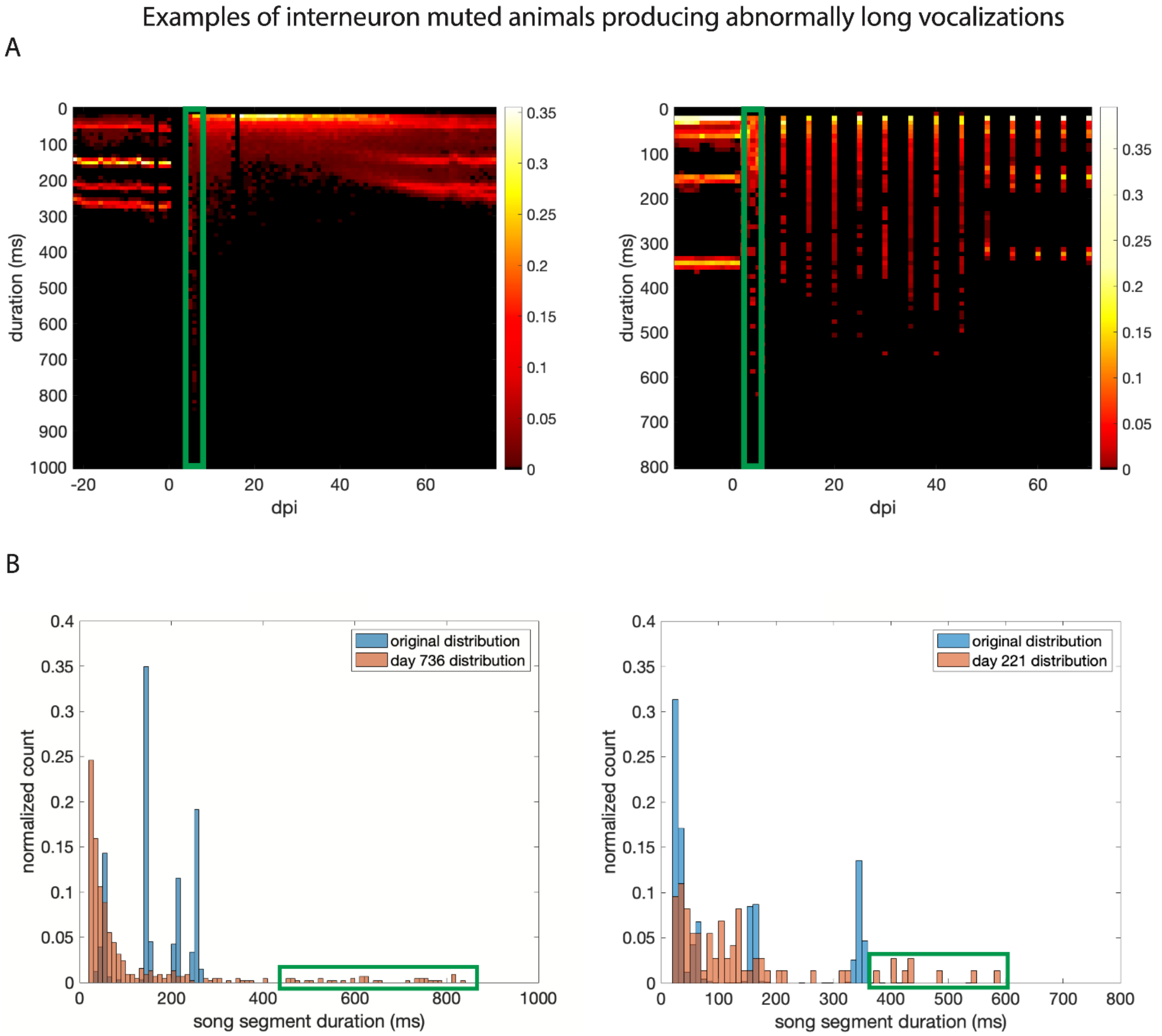

Supplementary Figure 1: *<u>Examples of abnormally long syllable lengths after injection of interneuron muting virus in two animals.</u>* ***A*** *Syllable length durations for the length of song degradation and recovery. The Y axis depicts the length of the syllables in milliseconds plotted over days post-injection (dpi) of either TeNT virus. TeNT-treated animals displayed a short period during which some vocalizations were of length not observed in normal animals and eventually became highly variable and shorter (shifts to shorter length sounds). The green rectangle highlights the day post-injection portrayed in B for each animal.* ***B*** *Histogram of syllable durations (blue trace is before injection of virus, orange trace is after injection of virus). The green rectangle highlights vocalizations of abnormal length.*

bioRxiv preprint doi: https://doi.org/10.1101/2023.05.17.541057; this version posted December 15, 2023. The copyright holder for this preprint (which was not certified by peer review) is the author/funder, who has granted bioRxiv a license to display the preprint in perpetuity. It is made available under aCC-BY 4.0 International license.

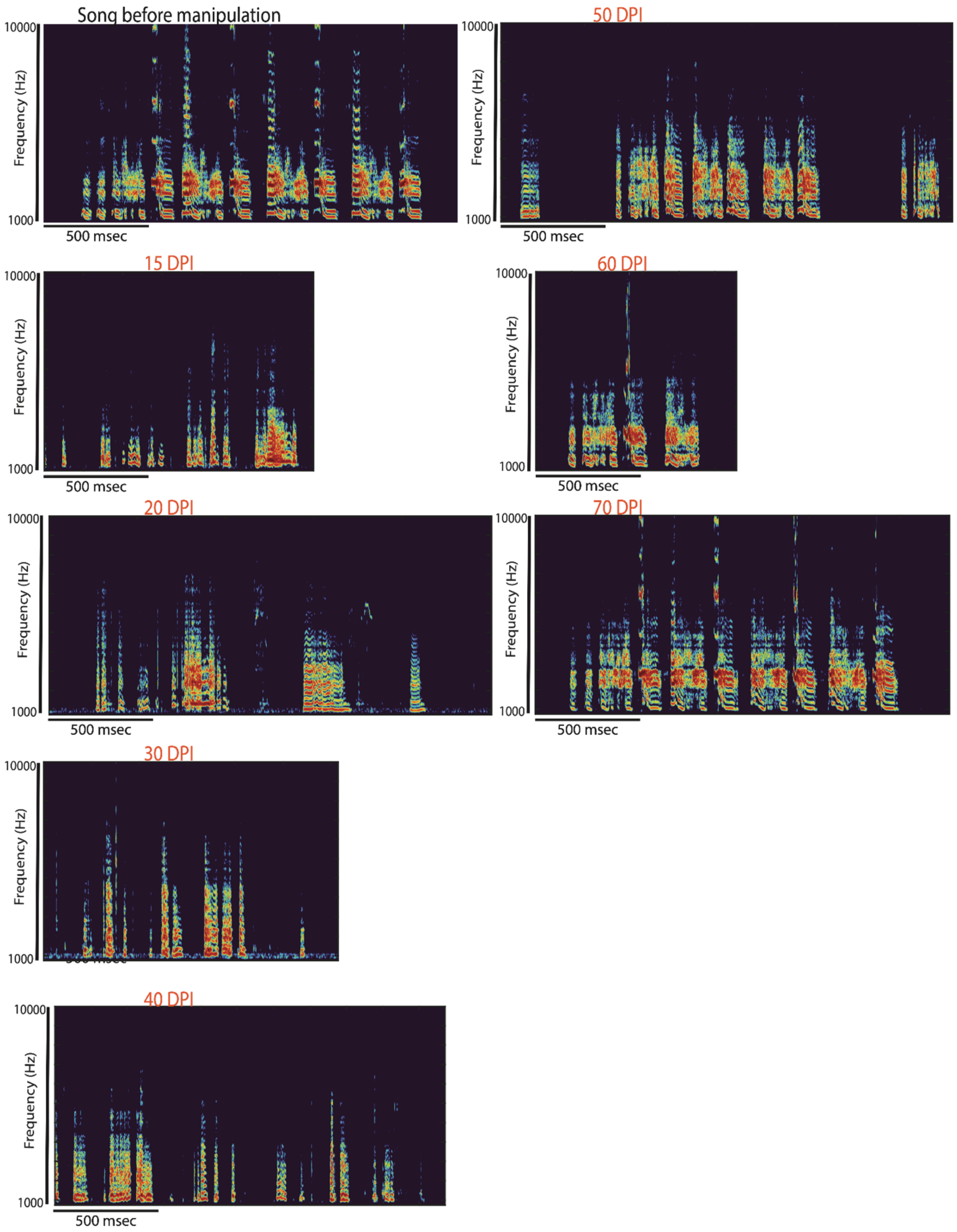

Figure legend on next page.

bioRxiv preprint doi: https://doi.org/10.1101/2023.05.17.541057; this version posted December 15, 2023. The copyright holder for this preprint (which was not certified by peer review) is the author/funder, who has granted bioRxiv a license to display the preprint in perpetuity. It is made available under aCC-BY 4.0 International license.

Supplementary Figure 2: *Example spectrograms of a TeNT-treated animal (B138) during song degradation and recovery. Vocalizations between 15 dpi and 30 dpi were much shorter than the first long syllables shown in Figure 1 A at 5 dpi.*

bioRxiv preprint doi: https://doi.org/10.1101/2023.05.17.541057; this version posted December 15, 2023. The copyright holder for this preprint (which was not certified by peer review) is the author/funder, who has granted bioRxiv a license to display the preprint in perpetuity. It is made available under aCC-BY 4.0 International license.

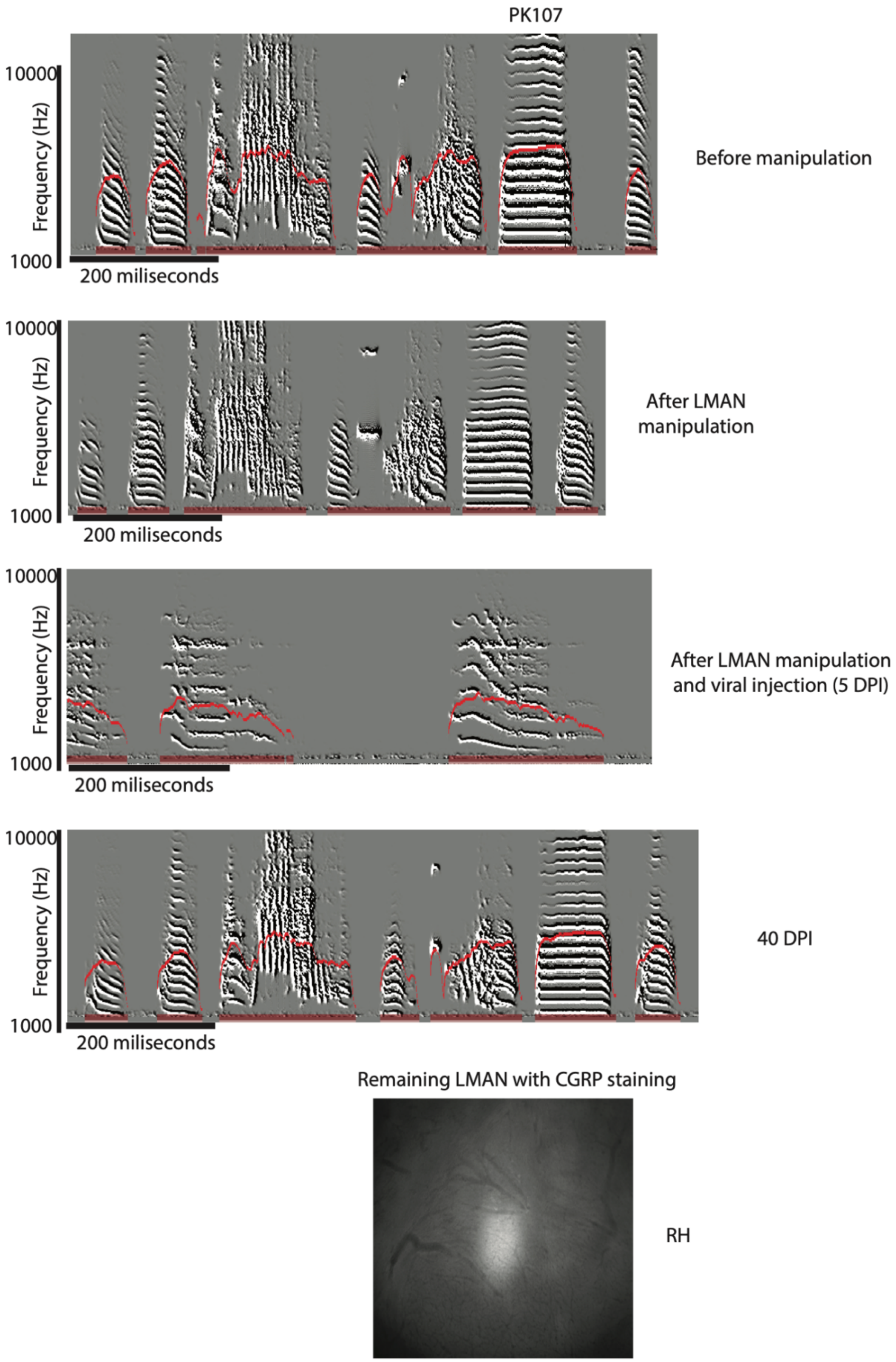

Figure legend on next page.

bioRxiv preprint doi: https://doi.org/10.1101/2023.05.17.541057; this version posted December 15, 2023. The copyright holder for this preprint (which was not certified by peer review) is the author/funder, who has granted bioRxiv a license to display the preprint in perpetuity. It is made available under aCC-BY 4.0 International license.

Supplementary Figure 3: *Song degradation and recovery after chronic removal of inhibition in an animal without LMAN. Spectrograms are showing the song of the animal before and after LMAN lesion at 5 and 40 days post viral injection (dpi). The histology image shows the amount of LMAN left (based on CGRP staining) in the right hemisphere (RH).*

bioRxiv preprint doi: https://doi.org/10.1101/2023.05.17.541057; this version posted December 15, 2023. The copyright holder for this preprint (which was not certified by peer review) is the author/funder, who has granted bioRxiv a license to display the preprint in perpetuity. It is made available under aCC-BY 4.0 International license.

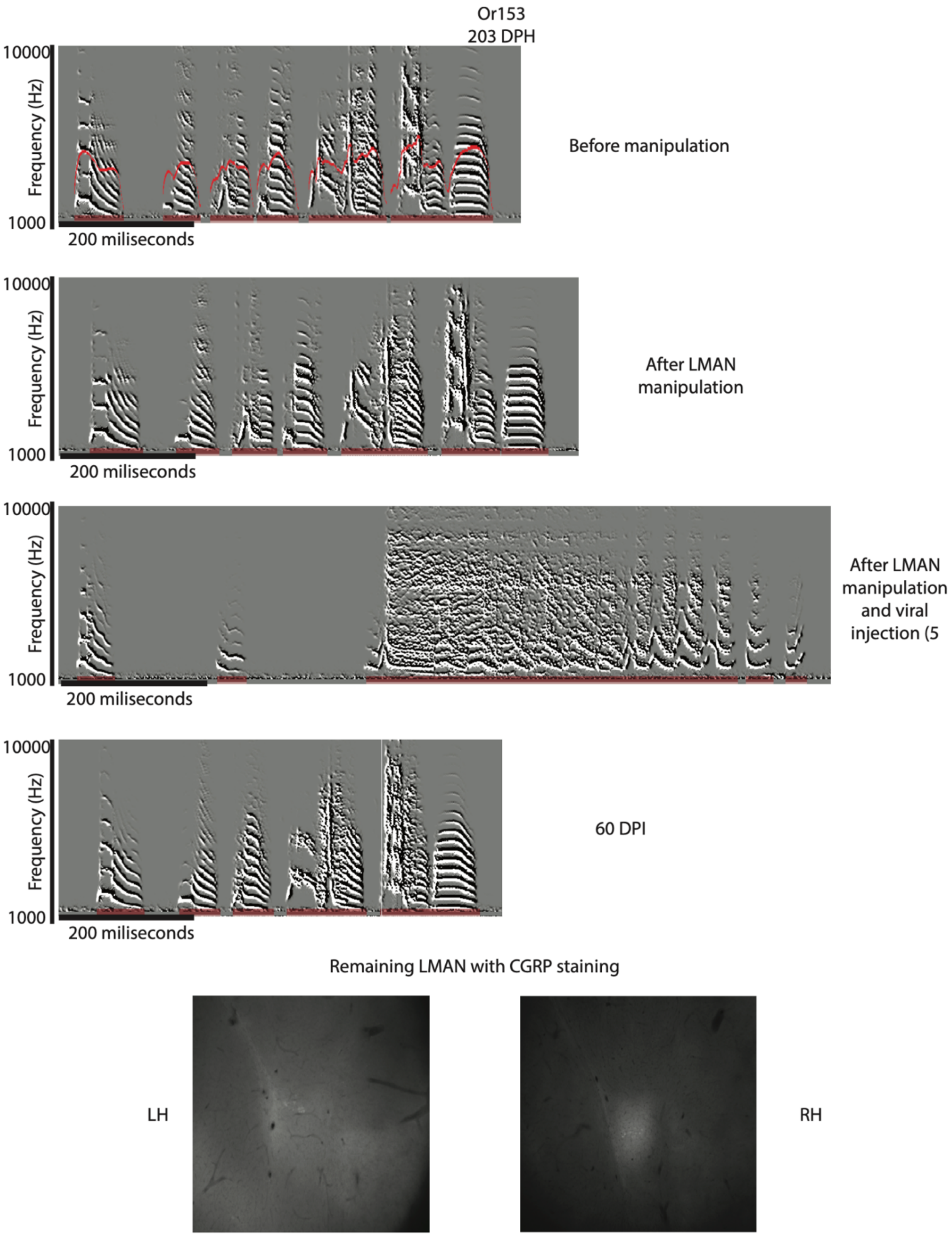

Figure legend on next page.

bioRxiv preprint doi: https://doi.org/10.1101/2023.05.17.541057; this version posted December 15, 2023. The copyright holder for this preprint (which was not certified by peer review) is the author/funder, who has granted bioRxiv a license to display the preprint in perpetuity. It is made available under aCC-BY 4.0 International license.

Supplementary Figure 4: *Song degradation and recovery after chronic removal of inhibition in an animal without LMAN. Spectrograms are showing the song of the animal before and after LMAN lesion at 5 and 60 days post viral injection (dpi). The histology images indicate the amount of LMAN left (based on CGRP staining) in the left (LH) and right (RH) hemispheres.*

bioRxiv preprint doi: https://doi.org/10.1101/2023.05.17.541057; this version posted December 15, 2023. The copyright holder for this preprint (which was not certified by peer review) is the author/funder, who has granted bioRxiv a license to display the preprint in perpetuity. It is made available under aCC-BY 4.0 International license.

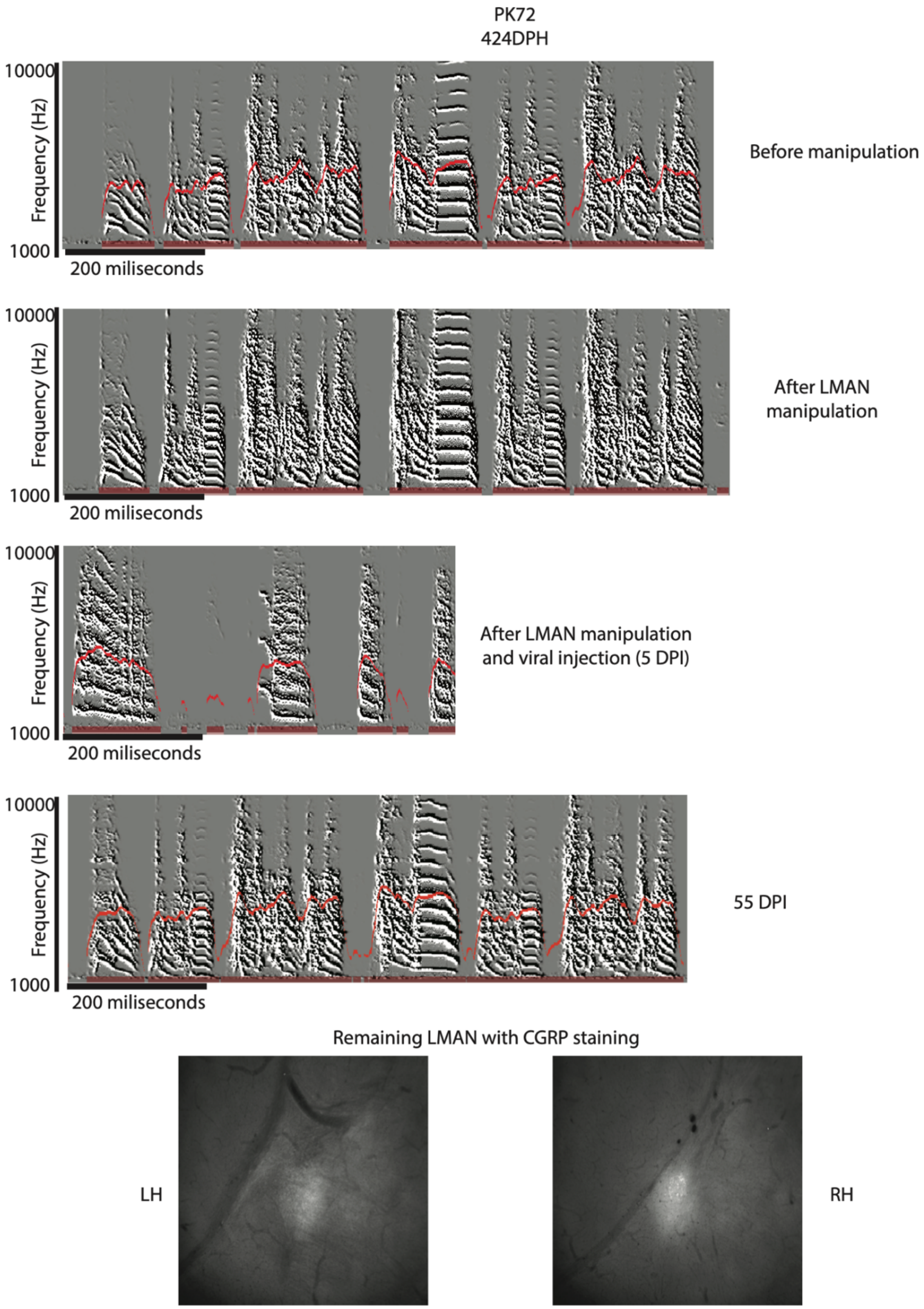

Figure legend on next page.

bioRxiv preprint doi: https://doi.org/10.1101/2023.05.17.541057; this version posted December 15, 2023. The copyright holder for this preprint (which was not certified by peer review) is the author/funder, who has granted bioRxiv a license to display the preprint in perpetuity. It is made available under aCC-BY 4.0 International license.

Supplementary Figure 5: *Song degradation and recovery after chronic removal of inhibition in an animal without LMAN. Spectrograms are showing the song of the animal before and after LMAN lesion at 5 and 55 days after viral injection (dpi). The histology images indicate the amount of LMAN left (based on CGRP staining) in the left (LH) and right (RH) hemispheres.*

bioRxiv preprint doi: https://doi.org/10.1101/2023.05.17.541057; this version posted December 15, 2023. The copyright holder for this preprint (which was not certified by peer review) is the author/funder, who has granted bioRxiv a license to display the preprint in perpetuity. It is made available under aCC-BY 4.0 International license.

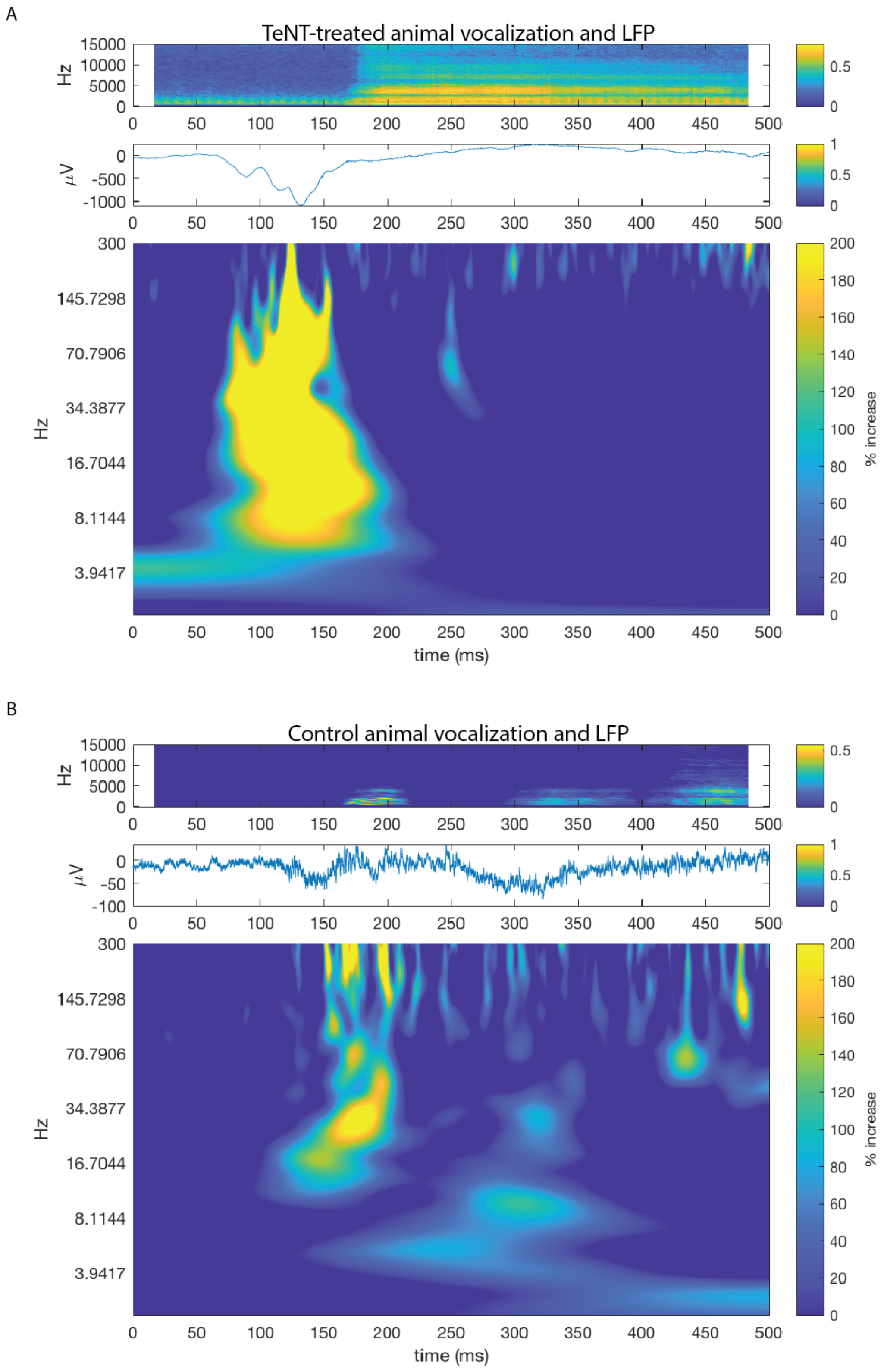

Figure legend on next page.

bioRxiv preprint doi: https://doi.org/10.1101/2023.05.17.541057; this version posted December 15, 2023. The copyright holder for this preprint (which was not certified by peer review) is the author/funder, who has granted bioRxiv a license to display the preprint in perpetuity. It is made available under aCC-BY 4.0 International license.

Supplementary Figure 6: *<u>Example electrophysiology traces averaged over 5 instances of normal or 5 instances of degraded vocalizations.</u>* ***A*** *Averaged spectrogram of degraded vocalization (n=5) 5 days post-electrode-implantation in a chronically recorded TeNT-treated animal. The plot below the spectrogram shows the raw averaged trace of extracellular recording. Below the raw trace is the averaged continuous wavelet transform of the local field potentials (LFP, 1-300Hz). The plots show a large deflection event (similar to those seen during lights-off in Figures 2 and 3) right before the onset of the vocalization in the TeNT-treated animal.* ***B*** *Averaged song spectrogram (n=5) 5 days post-electrode-implantation from a chronically recorded control animal. The plot below the spectrogram shows the raw averaged trace of extracellular recordings. Below the raw trace is the averaged continuous wavelet transform of the local field potentials (LFP, 1-300 Hz). The averaged control song shows more and smaller amplitude deflections mostly during the vocalization compared to the TeNT-treated vocalization.*

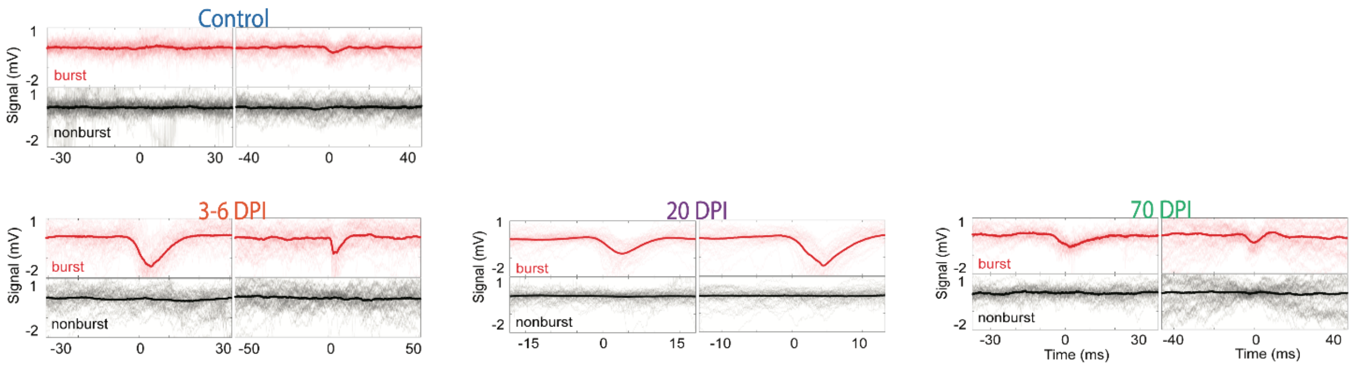

Supplementary Figure 7: *<u>Example traces of raw deflections in the acute Neuropixel recordings during lights-off periods.</u> Control animals barely showed any visible deflection events, while TeNT-treated animals (example traces shown at 3-6, 20, and 70 dpi) displayed large amplitude voltage deflection events.*

bioRxiv preprint doi: https://doi.org/10.1101/2023.05.17.541057; this version posted December 15, 2023. The copyright holder for this preprint (which was not certified by peer review) is the author/funder, who has granted bioRxiv a license to display the preprint in perpetuity. It is made available under aCC-BY 4.0 International license.

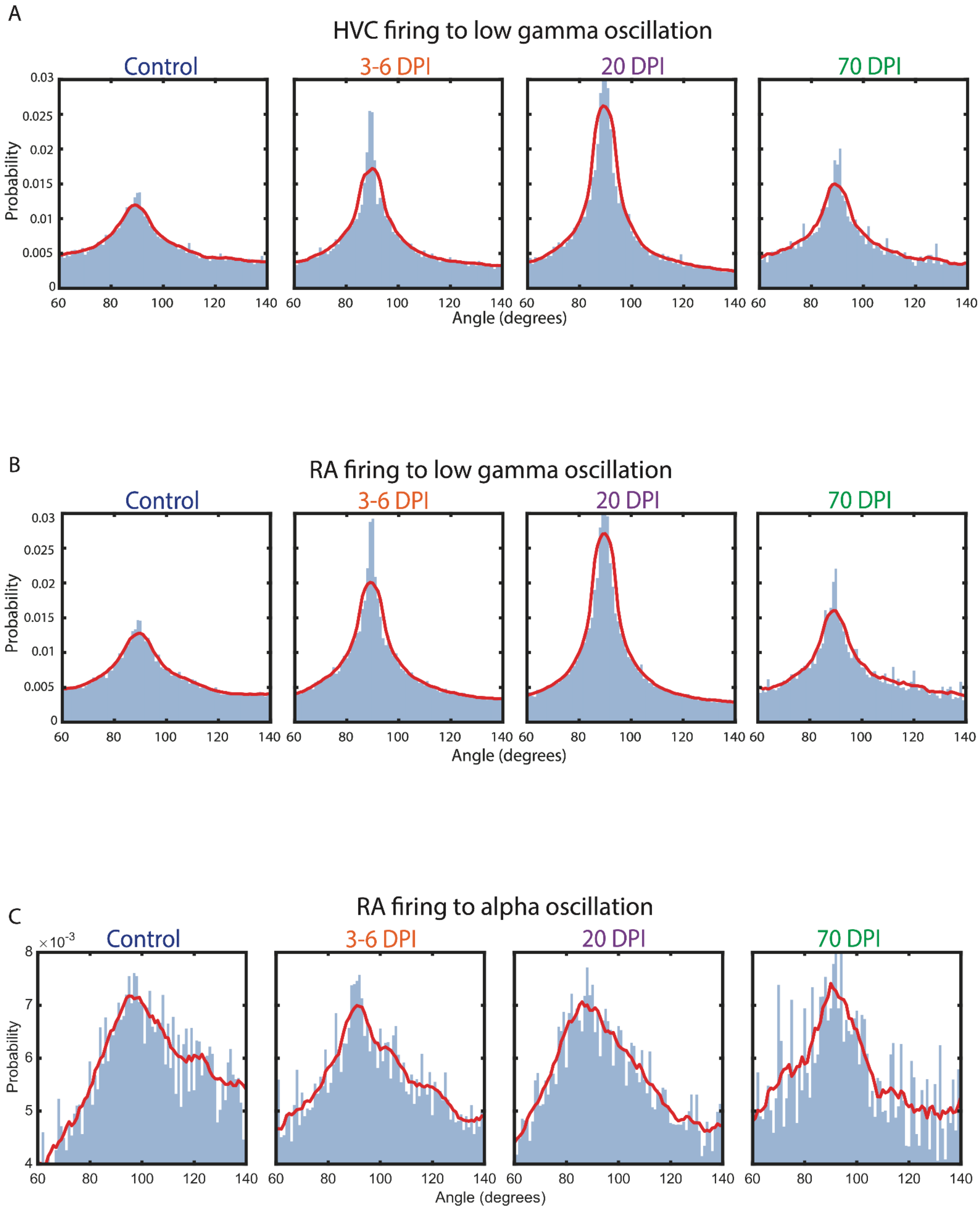

Figure legend on next page.

bioRxiv preprint doi: https://doi.org/10.1101/2023.05.17.541057; this version posted December 15, 2023. The copyright holder for this preprint (which was not certified by peer review) is the author/funder, who has granted bioRxiv a license to display the preprint in perpetuity. It is made available under aCC-BY 4.0 International license.

Supplementary Figure 8: *Relationship between alpha or gamma oscillations during lights-off voltage deflection events and local neuronal firing in HVC and RA from the acute NPIX recordings* ***A*** *The normalized probability distribution of neurons locally within HVC fire during a specific phase (angle) of the gamma (30-40 Hz) oscillations extracted from the LFP signal of the averaged deflection events at 3-6 dpi (n=4 animals), 20 dpi (n=4 animals), 70 dpi (n =2 animals that recovered their song by then). There was a slight change in local neuronal firing to the angle and locking precision to gamma oscillations that resembled control by 70 dpi.* ***B*** *Normalized probability distribution of neurons locally within RA fire during a specific phase (angle) of the alpha (1-10 Hz) oscillations in HVC extracted from the LFP signal of the averaged deflection events at 3-6 dpi, 20, 70 dpi. No change in RA spontaneous neuronal firing to alpha oscillations in HVC during the deflection events over the course of the manipulation.* ***C*** *Normalized probability distribution of neurons locally within RA fire during a specific phase (angle) of the gamma (30-40 Hz) oscillations in HVC extracted from the LFP signal of the averaged deflection events at 3-6, 20, and 70 dpi. We observed no change in RA spontaneous neuronal firing to the gamma oscillations in HVC during the deflection events over the course of the manipulation.*

bioRxiv preprint doi: https://doi.org/10.1101/2023.05.17.541057; this version posted December 15, 2023. The copyright holder for this preprint (which was not certified by peer review) is the author/funder, who has granted bioRxiv a license to display the preprint in perpetuity. It is made available under aCC-BY 4.0 International license.

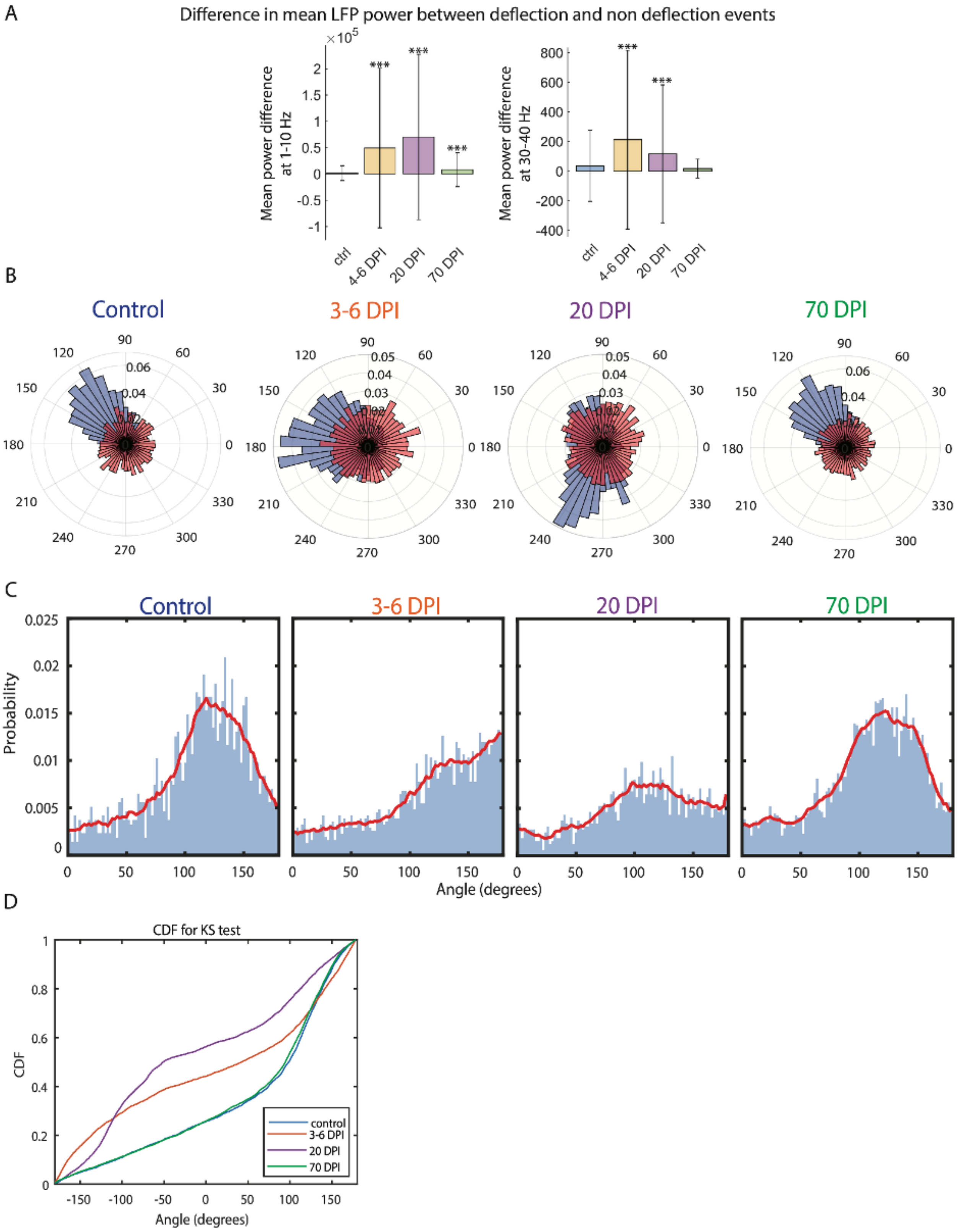

Figure legend on next page.

bioRxiv preprint doi: https://doi.org/10.1101/2023.05.17.541057; this version posted December 15, 2023. The copyright holder for this preprint (which was not certified by peer review) is the author/funder, who has granted bioRxiv a license to display the preprint in perpetuity. It is made available under aCC-BY 4.0 International license.

Supplementary Figure 9: *Quantification of the angle relationship between alpha (1-10 Hz) and gamma (30-40 Hz) frequencies during deflection events in control and TeNT-treated animals during acute head-fixed recordings.* ***A****: The average difference in power (at alpha, 1-10 Hz, and low gamma 30-40 Hz frequency ranges) between voltage deflection and non-deflection events in control, 3-6, 20, 70 dpi. The power content in the alpha range increased in a statistically significant way (Wilcoxon, rank sum test) between control and 3-6 (p=2.9*10^-36), 20 (p=8.6*10^-167), 70 (p=5.4*10^-4) dpi animals. However, the increase in power between control and 3-6 (p=2.2*10^-37), 20 (p=6.3*10^-27) dpi is statistically significant but returns to control level by 70 (p=0.37) dpi. The stars above the bar plots (*) indicate statistical significance (* : p < 0.005, ** : p< 0.01, *** : p < 0.001).* ***B****: The polar histograms of the angle of the low gamma oscillations (1-10Hz) at the maximum amplitude of the gamma oscillation (30-40 Hz) during deflection events. The red distribution represents a randomly shuffled dataset, while the blue is the true distribution of angles in control (n=3), and TeNT-treated animals at 3-6 dpi (n=4), 20 dpi (n=4) and 70 dpi (n=4) during deflection events.* ***C****: Relationship of alpha and low gamma oscillations during deflection events in control (n=3), 3-6 (n=4), 20 (n=4), and 70 (n=2 animals) dpi animals (over animals and conditions). The probability distribution of a specific angle of the low-frequency oscillation at the maximum amplitude of the gamma oscillation.* ***D****: The results of the Kolgomorov-Smirnov test on the cumulative density function (CDF) to assess if the change in probability distribution shown in C is statistically significant from control distributions at 3-6, 20, and 70 dpi. The purple (20 dpi) and orange (3-7 dpi) distributions differ significantly from the blue control and the 70 dpi green distributions. The 70 dpi population is not significantly different from the control group.*

bioRxiv preprint doi: https://doi.org/10.1101/2023.05.17.541057; this version posted December 15, 2023. The copyright holder for this preprint (which was not certified by peer review) is the author/funder, who has granted bioRxiv a license to display the preprint in perpetuity. It is made available under aCC-BY 4.0 International license.

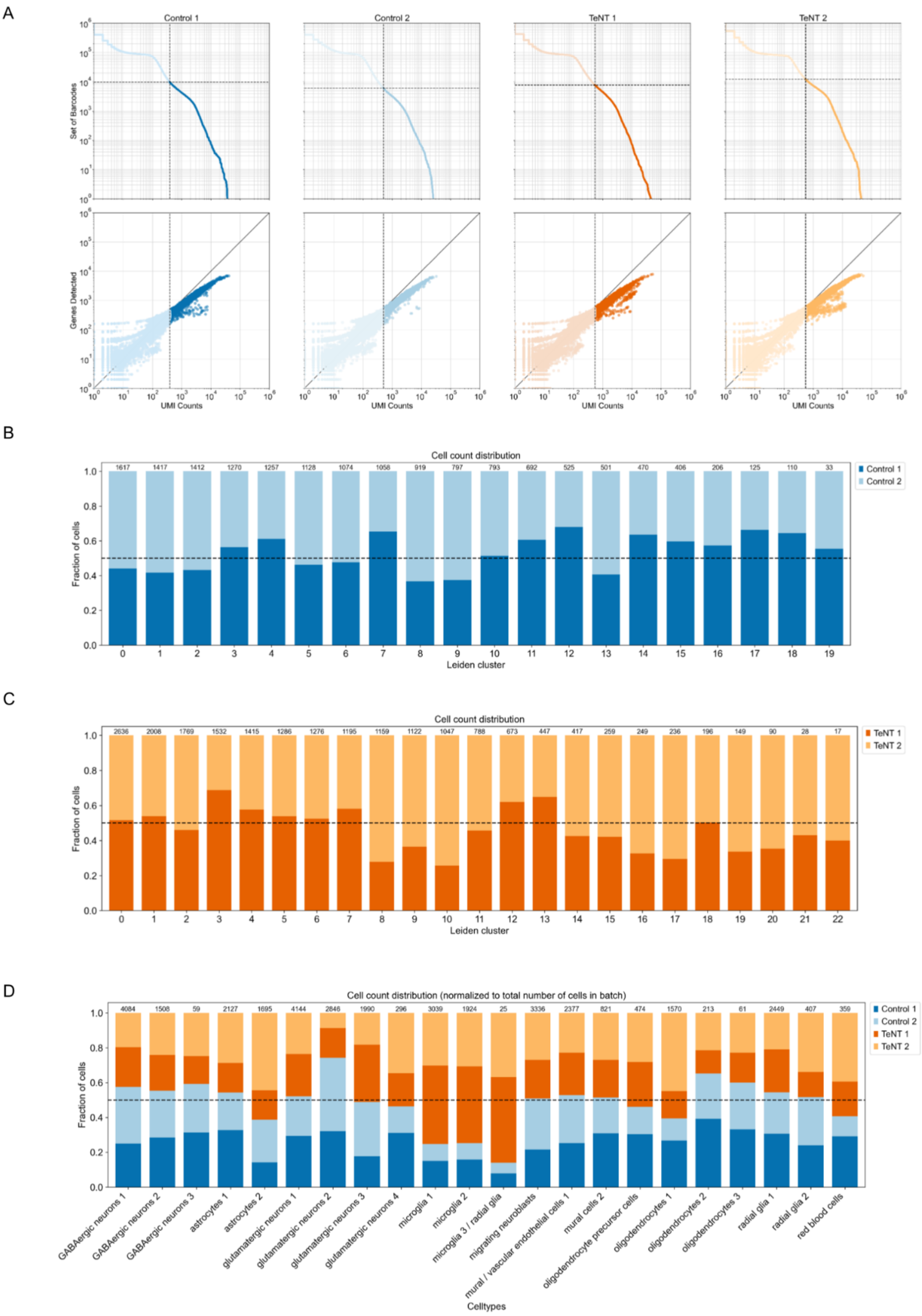

Figure legend on next page.

bioRxiv preprint doi: https://doi.org/10.1101/2023.05.17.541057; this version posted December 15, 2023. The copyright holder for this preprint (which was not certified by peer review) is the author/funder, who has granted bioRxiv a license to display the preprint in perpetuity. It is made available under aCC-BY 4.0 International license.

Supplementary Figure 10: *Quality control of the single-cell RNA sequencing HVC datasets from control and TeTN-treated animals at 25 days post-injection (dpi).* ***A*** *"Knee plots" showing the set of barcodes (top row) and number of genes detected (bottom row) over UMI counts. The dashed lines depict the quality filtering cutoff.* ***B-C*** *Barplot depicting the fraction of cells from each replicate per cluster for control (B) and TeNT (C), normalized (by dividing) to the total number of cells in each replicate. Control and TeNT datasets were clustered separately using the Leiden algorithm. The equal distribution of replicates across the clusters suggests that technical effects do not dominate the clusters. Thus, we did not perform batch correction. The numbers on top of the bars indicate the total number of cells in each cluster.* ***D*** *Barplot depicting the fraction of cells from each dataset in the cell type clusters obtained after jointly clustering the control and TeNT datasets. The numbers on top of the bars indicate the total number of cells in each cluster.*

bioRxiv preprint doi: https://doi.org/10.1101/2023.05.17.541057; this version posted December 15, 2023. The copyright holder for this preprint (which was not certified by peer review) is the author/funder, who has granted bioRxiv a license to display the preprint in perpetuity. It is made available under aCC-BY 4.0 International license.

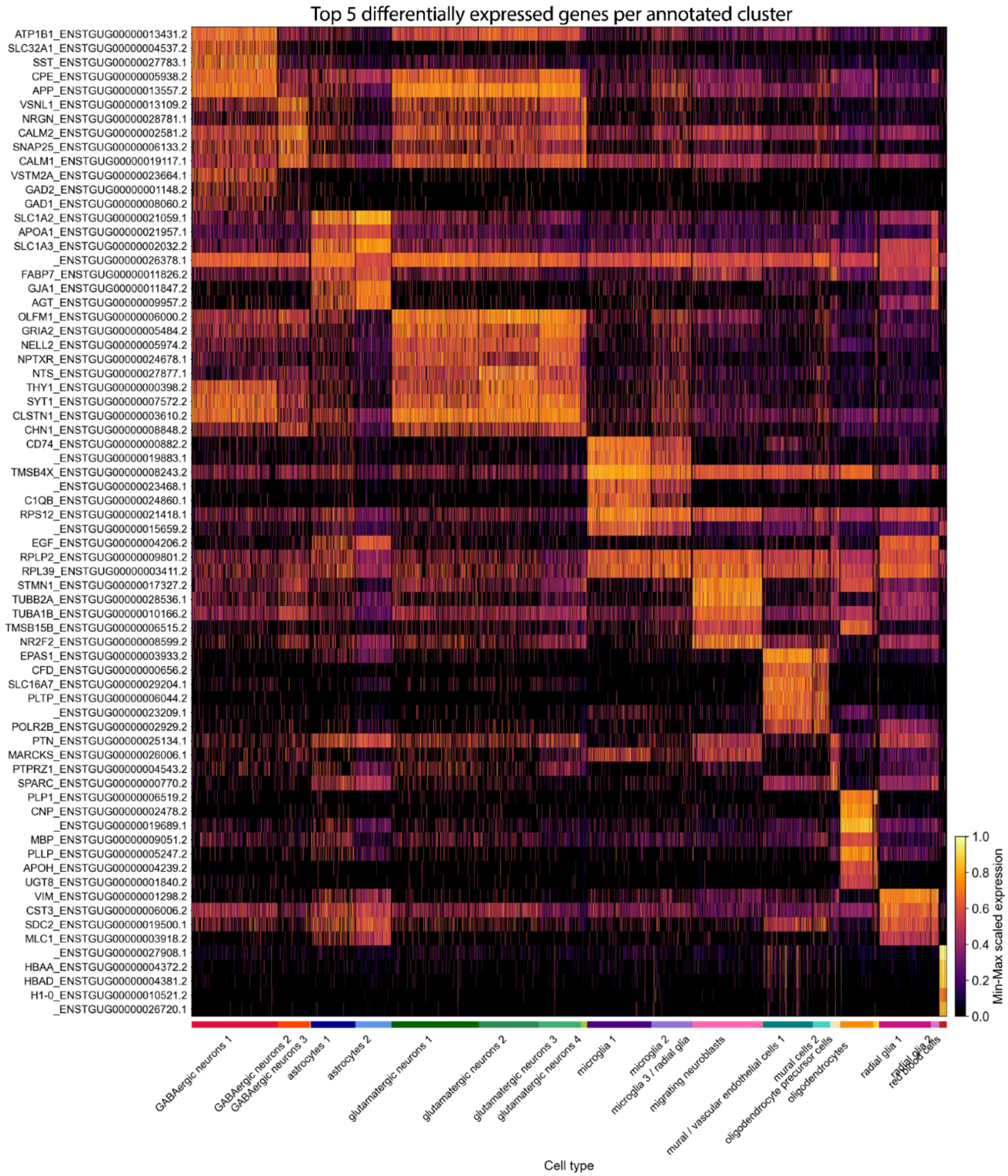

Supplementary Figure 11: *<u>Heatmap of top 5 differentially expressed genes per annotated cell type/cluster obtained by single-cell RNA sequencing of HVC from control and TeNT-treated birds at 25 dpi.</u> Differentially expressed genes between clusters were identified using Scanpy's rank_genes_groups (p values were computed using a t-test and were adjusted with the Bonferroni method for multiple testing. They were then confirmed by comparison to p values generated with the nonparametric Wilcoxon test with Bonferroni correction). The heatmap depicts the min-max scaled expression for each gene.*

bioRxiv preprint doi: https://doi.org/10.1101/2023.05.17.541057; this version posted December 15, 2023. The copyright holder for this preprint (which was not certified by peer review) is the author/funder, who has granted bioRxiv a license to display the preprint in perpetuity. It is made available under aCC-BY 4.0 International license.

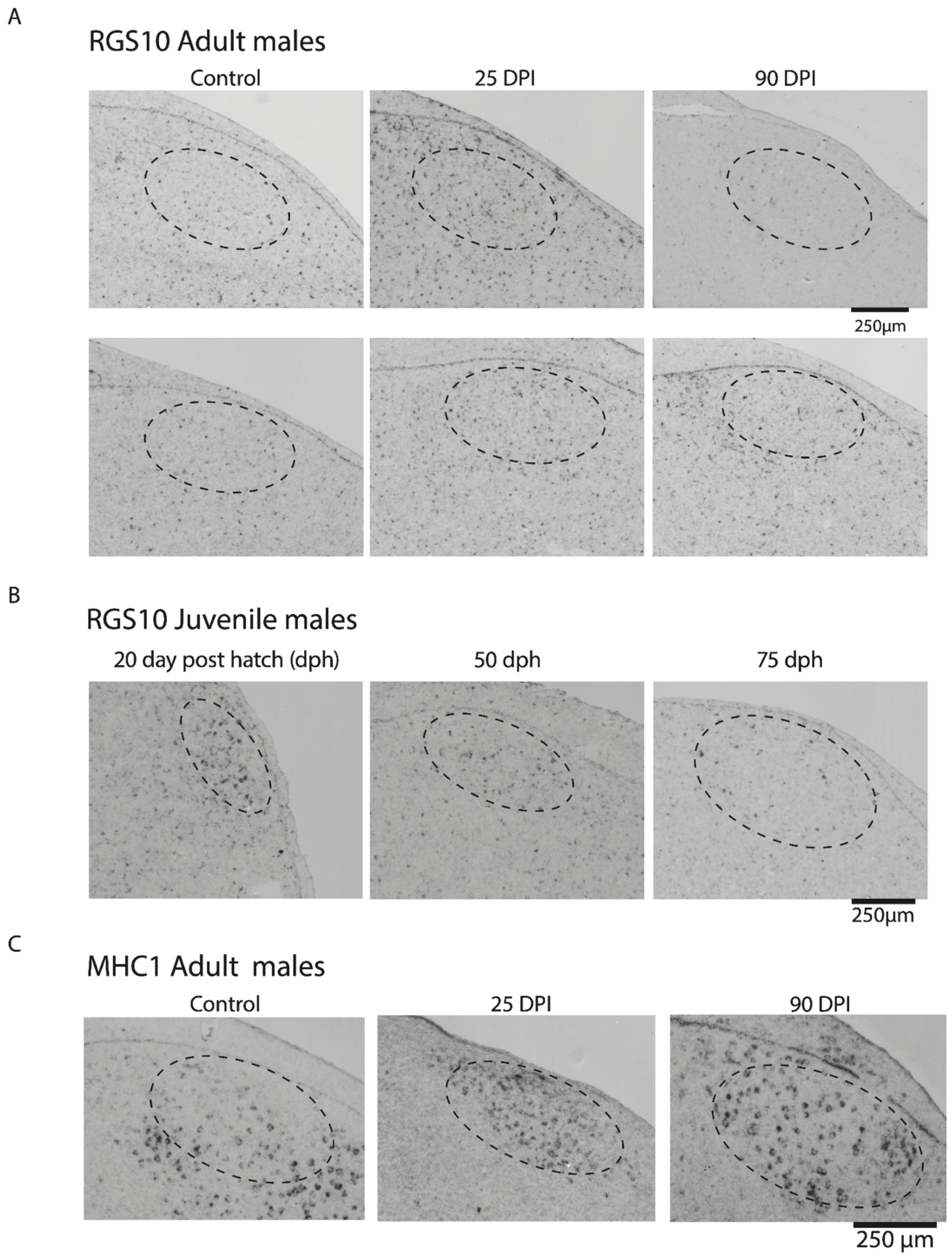

Supplementary Figure 12: *In situ hybridization of microglia marker gene RGS10 in adult male control, TeNT-treated and juvenile male HVC & MHC1 gene in adult male control and TeNT-treated HVC.* ***A*** *Histological sections of HVC (in control and TeNT-treated animals at 25 and 90 dpi) after in situ hybridization of RNA probes for RGS10 (a gene marker for microglia).* ***B*** *Histological sections of HVC in naive juvenile males (at 20, 50, and 75 days post-hatching (dph)) after in situ hybridization of RNA probes for RGS10.* ***C*** *Histological sections of HVC (from control and TeNT-treated animals at 25 and 90 dpi) after in situ hybridization of RNA probes for MHC1. Black/darker dots indicate enzyme reactions resulting in successful probe localization and suggest target gene expression.*

bioRxiv preprint doi: https://doi.org/10.1101/2023.05.17.541057; this version posted December 15, 2023. The copyright holder for this preprint (which was not certified by peer review) is the author/funder, who has granted bioRxiv a license to display the preprint in perpetuity. It is made available under aCC-BY 4.0 International license.

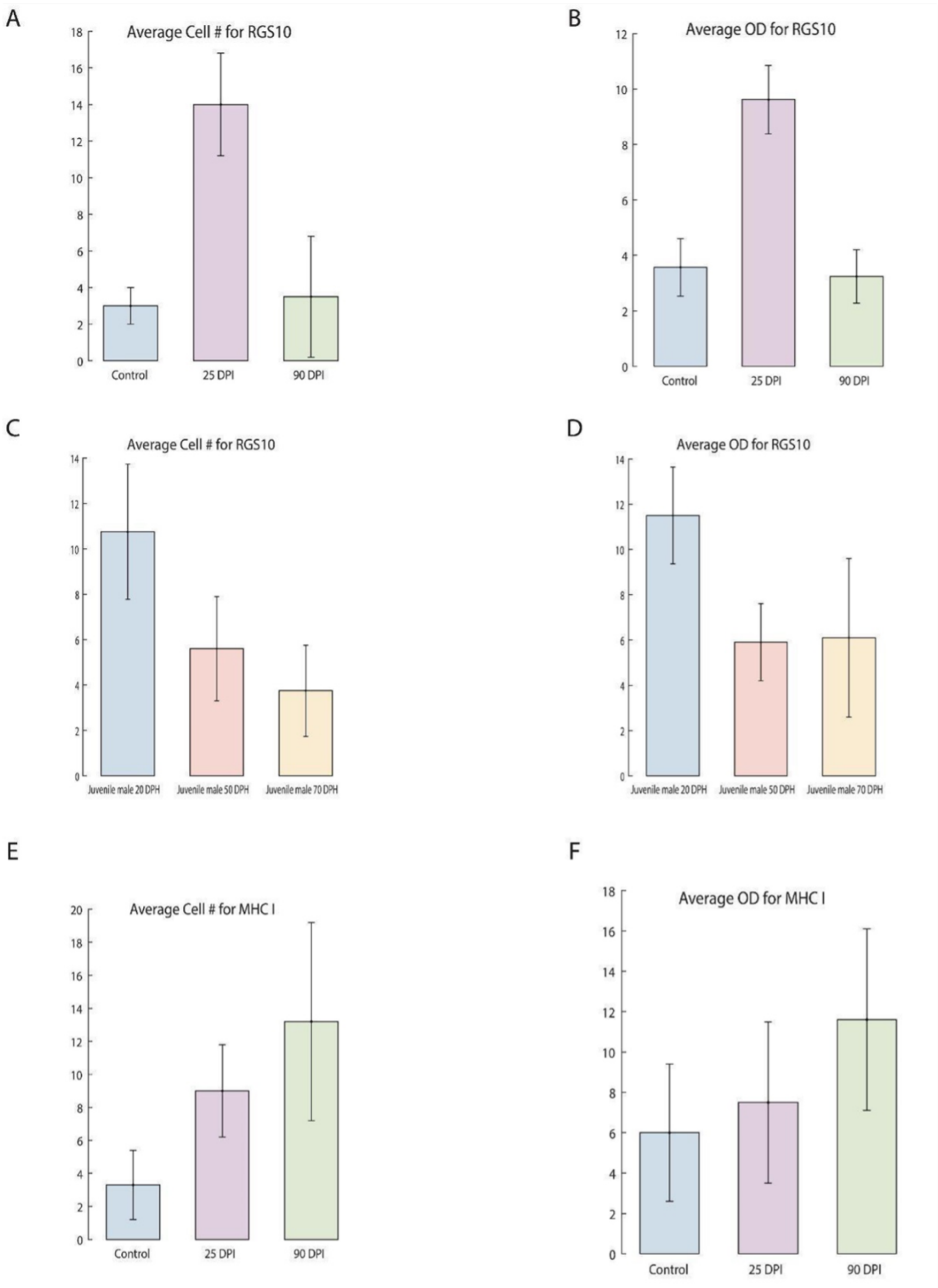

Supplementary Figure 13: *Quantification of the in situ hybridization against microglia marker gene RGS10 in adult male control, TeNT-treated and juvenile male HVC; and MHC1 in adult male control, TeNT-treated animals.* ***A-B*** *Quantification of the in situ hybridization for RGS10 between control (n=4 animals) and TeNT-treated animals at 25 dpi (n=4) and 90 dpi (n=4).* ***C-D*** *Quantification of the in situ hybridization for RGS10 between juvenile males at 20, 50, and 70 days post-hatching (dph) (n=4).* ***E-F*** *Quantification of the in situ hybridization for MHC1 between control (n=4) and TeNT-treated animals at 25 (n=4) and 90 dpi (n=4). Error bars represent standard deviation.*

bioRxiv preprint doi: https://doi.org/10.1101/2023.05.17.541057; this version posted December 15, 2023. The copyright holder for this preprint (which was not certified by peer review) is the author/funder, who has granted bioRxiv a license to display the preprint in perpetuity. It is made available under aCC-BY 4.0 International license.

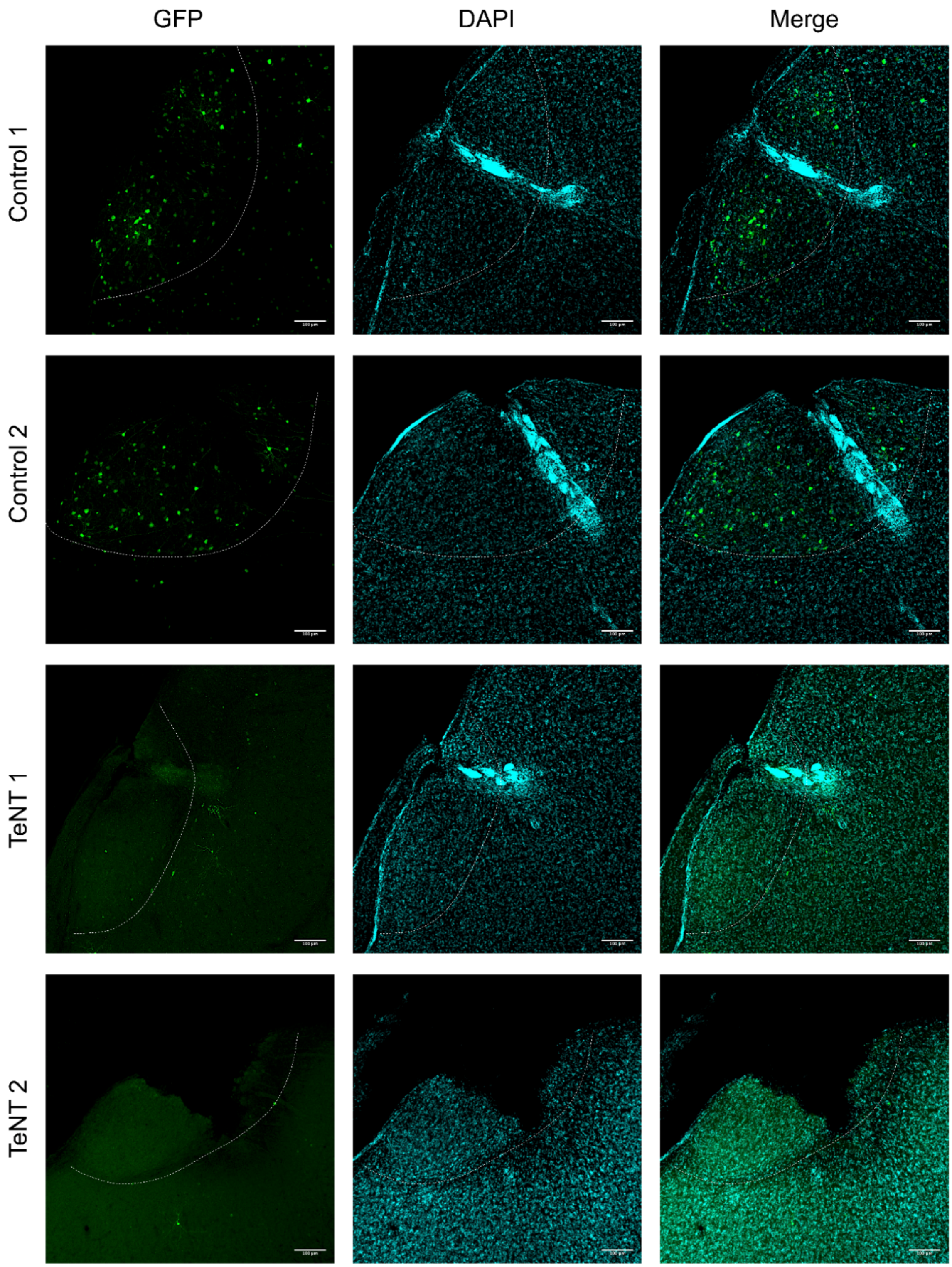

Supplementary Figure 14: *<u>Histology of electrode array location in HVC in the chronically implanted animals.</u> The white dotted line outlines HVC. Some sections display missing tissue due to the removal of the electrodes after perfusion of the animals. The stronger cyan signal indicates glial scar formation around the electrode array, which provides an approximation of the location of the electrodes. Electrodes located closer to the bottom of HVC close to the shelf were not used for analysis.*

bioRxiv preprint doi: https://doi.org/10.1101/2023.05.17.541057; this version posted December 15, 2023. The copyright holder for this preprint (which was not certified by peer review) is the author/funder, who has granted bioRxiv a license to display the preprint in perpetuity. It is made available under aCC-BY 4.0 International license.

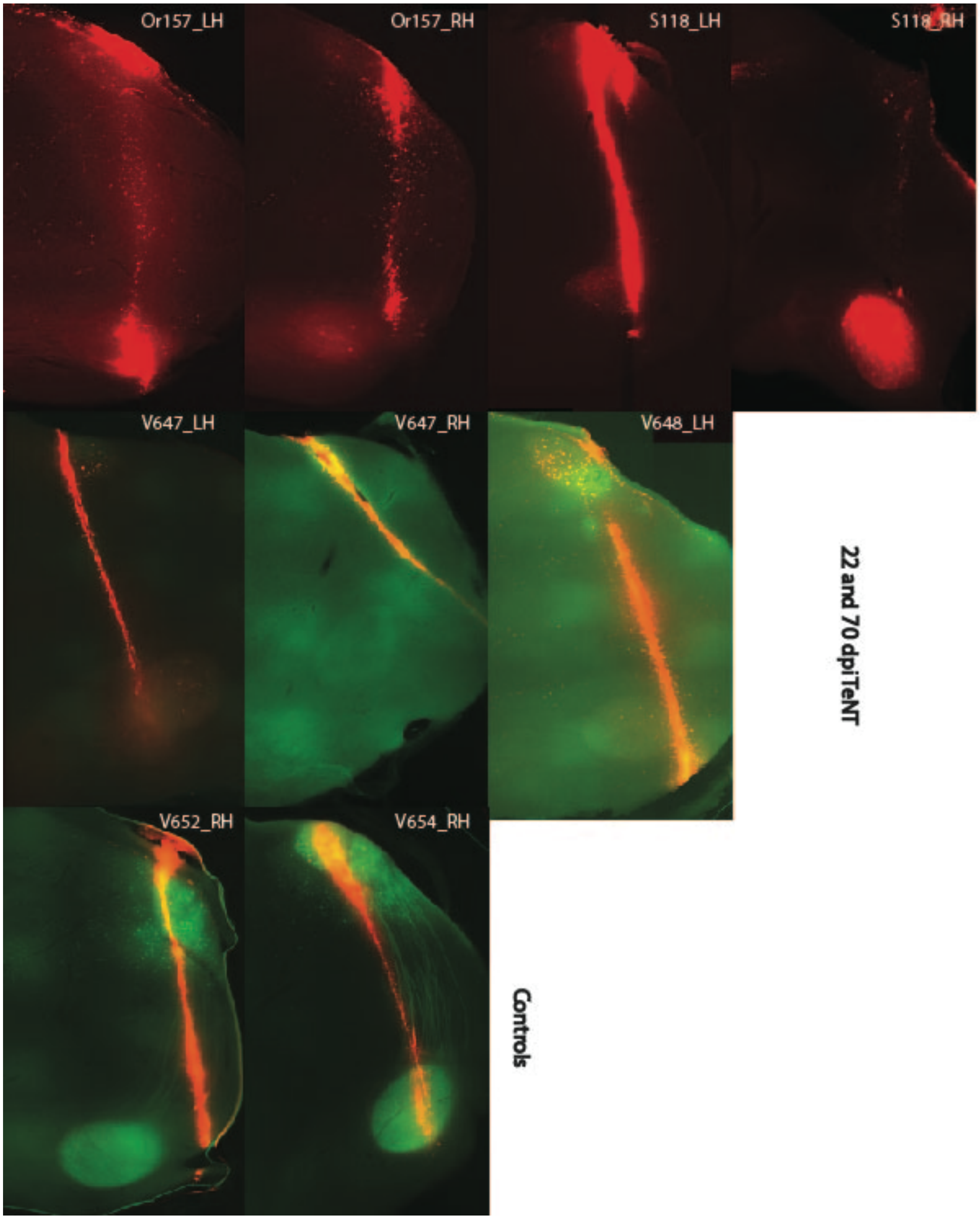

Supplementary Figure 15: *Histology to confirm the high-density silicone electrode location in the acute head-fixed animal recordings. The red trace represents the electrode location. The green trace represents the second*

bioRxiv preprint doi: https://doi.org/10.1101/2023.05.17.541057; this version posted December 15, 2023. The copyright holder for this preprint (which was not certified by peer review) is the author/funder, who has granted bioRxiv a license to display the preprint in perpetuity. It is made available under aCC-BY 4.0 International license.

electrode location in animals that were recorded twice, 40 days apart. The white labels represent the animal IDs. "LH" and "RH" stands for left and right hemisphere, respectively.

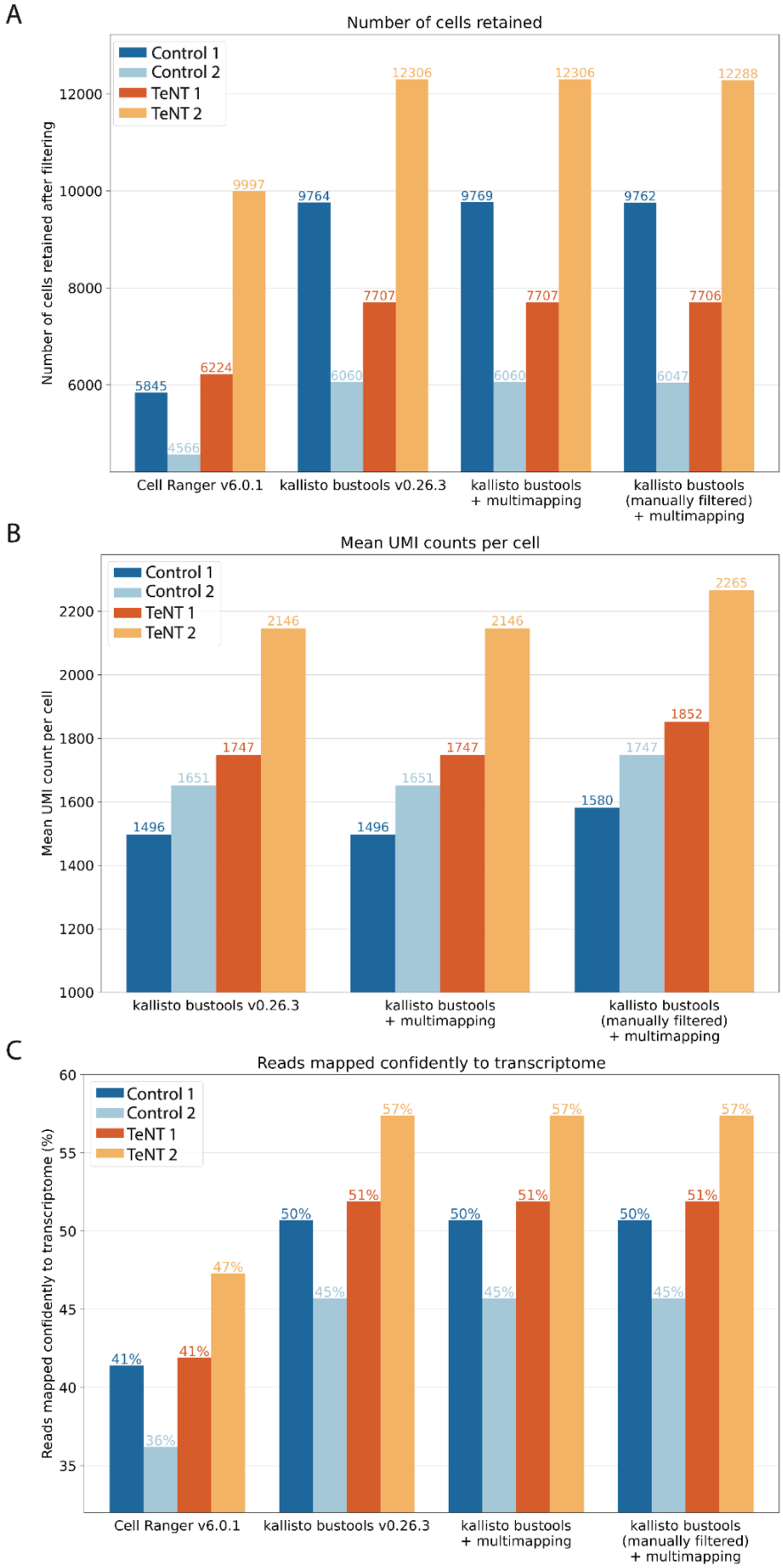

Figure legend on next page.

bioRxiv preprint doi: https://doi.org/10.1101/2023.05.17.541057; this version posted December 15, 2023. The copyright holder for this preprint (which was not certified by peer review) is the author/funder, who has granted bioRxiv a license to display the preprint in perpetuity. It is made available under aCC-BY 4.0 International license.

Supplementary Figure 16: *Comparison of different pre-processing methods for the HVC single-cell RNA sequencing datasets.* ***A*** *Number of cells retained after quality control for each dataset and alignment method.* ***B*** *Mean UMI counts per cell for each dataset and pre-processing method.* ***C*** *Percentage of reads confidently mapped to transcriptome for each pre-processing method.*

A

Deflection durations (half-widths) in ms (mean ± SD)

	3-5 DPI	15 DPI	30 DPI	45 DPI	60 DPI	75 DPI
Control animal 1 (OR295)	44.2083 ±16.0885	50.7321 ±21.5322	47.7079 ±16.4028	49.5889 ±18.5678	45.4170 ±15.4166	48.2883 ±18.5856
Control animal 2 (PK31)	43.9120 ±15.5607	46.5072 ±17.6237	36.7974 ±15.6453	42.4989 ±15.8985	37.8048 ±15.2497	35.3157 ±15.2297
TeNT-treated animal 1 (B138)	24.7074 ±11.0296	20.4568 ±5.7482	26.2938 ±10.4427	32.1460 ±13.1017	36.8212 ±15.3777	41.4122 ±15.9937
TeNT-treated animal 2 (OR296)	34.6744 ±10.7396	29.2852 ±8.3464	29.5713 ±9.5446	31.4171 ±11.5461	39.8151 ±10.5067	42.9507 ±14.0682

B

Deflection amplitudes in µV (mean ± SD)

	3-5 DPI	15 DPI	30 DPI	45 DPI	60 DPI	75 DPI
Control animal 1 (OR295)	-136.3911 ±28.7491	-155.0317 ±49.3434	-151.5708 ±35.8116	-149.0358 ±34.9365	-152.4109 ±33.1907	-150.5303 ±33.8846
Control animal 2 (PK31)	-111.9867 ±20.8258	-117.1694 ±26.5227	-133.9981 ±26.5174	-130.6166 ±25.9310	-138.6591 ±26.7510	-135.2756 ±25.9969
TeNT-treated animal 1 (B138)	-538.6617 ±307.8410	-816.7580 ±371.9481	-705.8811 ±267.1738	-422.1097 ±143.1100	-306.8821 ±101.9335	-261.2531 ±80.5379
TeNT-treated animal 2 (OR296)	-174.6613 ±39.6156	-251.7470 ±70.1546	-337.3158 ±94.5369	-525.5973 ±157.8933	-228.3434 ±48.9163	-162.6949 ±35.6879

Table legend on next page.

bioRxiv preprint doi: https://doi.org/10.1101/2023.05.17.541057; this version posted December 15, 2023. The copyright holder for this preprint (which was not certified by peer review) is the author/funder, who has granted bioRxiv a license to display the preprint in perpetuity. It is made available under aCC-BY 4.0 International license.

Supplementary Table 1: *Amplitudes and durations of the chronic voltage deflections measured throughout the recording.* ***A*** *Mean duration (calculated as the distance from the onset to the half-width point of the event) in ms of voltage deflection events with standard deviation, each row represents an event from one control (Or 295, PK31) or TeNT-treated (B138, Or296) animal. The data was sampled at 3-5, 15, 30, 45, 60, and 75 dpi.* ***B*** *Mean amplitudes (in µV) of voltage deflection events with standard deviation.*

Dataset (short name)	Species	Condition	Brain area	Replicate #	Technology	Pre-processing tool	# of cells retained after QC	Sequencing depth (number of reads processed)	Reads mapped confidently to transcriptome (%)	Mean UMI count per cell	Total UMI count
C1	Taeniopygia guttata	cag-neonGreen	HVC (both hemispheres)	1	10xv3	kallisto bustools	9,763	744,473,151	50.7	1580.8531	15,433,015
C2	Taeniopygia guttata	cag-neonGreen	HVC (both hemispheres)	2	10xv3	kallisto bustools	6,047	787,232,472	45.7	1747.639	10,568,132
E1	Taeniopygia guttata	dlx-TeNT-GFP	HVC (both hemispheres)	1	10xv3	kallisto bustools	7,706	867,768,600	51.9	1852.4215	14,274,784
E2	Taeniopygia guttata	dlx-TeNT-GFP	HVC (both hemispheres)	2	10xv3	kallisto bustools	12,288	810,253,355	57.4	2265.4258	27,837,586

Supplementary Table 2: *Overview of single-cell RNA sequencing datasets.*
